# Decreased CO_2_ saturation during circular breathwork supports emergence of altered states of consciousness

**DOI:** 10.1038/s44271-025-00247-0

**Published:** 2025-04-13

**Authors:** Martha N. Havenith, Max Leidenberger, Jelena Brasanac, Mafalda Corvacho, Inês Carmo Figueiredo, Leonie Schwarz, Malin Uthaug, Simona Rakusa, Marijan Bernardic, Liliana Vasquez-Mock, Sergio Pérez Rosal, Robin Carhart-Harris, Stefan M. Gold, Henrik Jungaberle, Andrea Jungaberle

**Affiliations:** 1 Zero-Noise Lab, Ernst Strüngmann Institute for Neuroscience, Frankfurt a.M, Germany; 2 MIND Foundation, Berlin, Germany; 3 Charité—Universitätsmedizin Berlin, Department of Psychiatry and Neuroscience, Campus Benjamin Franklin, Berlin, Germany; 4 Charité—Universitätsmedizin Berlin, Medical Department, Psychosomatic Medicine, Campus Benjamin Franklin, Berlin, Germany; 5 Department of Neuropsychology and Psychopharmacology, Faculty of Psychology and Neuroscience, Maastricht University, Maastricht, Netherlands; 6 Somnivore Pty. Ltd., Bacchus Marsh, VIC Australia; 7 The Centre for Psychedelic Research, Department of Brain Sciences, Faculty of Medicine, Imperial College London, London, UK; 8 Universitätsklinikum Ruppin-Brandenburg, Berlin, Germany; 9 Weill Institute for Neurosciences, University of California San Francisco, San Francisco, CA USA; 10 Sandler Neurosciences Center, University of California San Francisco, San Francisco, CA USA; 11 Institute of Neuroimmunology and Multiple Sclerosis (INIMS), Universitätsklinikum Hamburg-Eppendorf, Hamburg, Germany; 12 German Center for Mental Health (DZPG), Campus Charité Mitte, Berlin, Germany; 13 OVID Clinics, Berlin, Germany

**Keywords:** Consciousness, Human behaviour, Human behaviour

## Abstract

Altered states of consciousness (ASCs), induced e.g. during psychedelic-assisted therapy, show potential to treat prevalent mental health disorders like depression and posttraumatic stress disorder. However, access to such treatments is restricted by legal, medical, and financial barriers. Circular breathwork may present a non-pharmacological and hence more accessible alternative to engage similar therapeutic processes. Scientific studies of breathwork are only just emerging and its physiological and psychological mechanisms are largely unknown. Here, we track physiological and experiential dynamics throughout a breathwork session, comparing two forms of breathwork: Holotropic and Conscious-Connected breathwork. We show that a reduction in end-tidal CO_2_ pressure due to deliberate hyperventilation is significantly correlated to ASC onset (*r* = -0.46; *p* < 0.001). Based on standard questionnaires (MEQ-30 and 11-DASC), the ASCs evoked by breathwork resembled those produced by psychedelics across several experiential domains such as ego dissolution, and their depth predicted psychological and physiological follow-on effects, including improved well-being and reduced depressive symptoms. Further analysis showed that different breathwork approaches produced highly similar outcomes. Our findings identify physiological boundary conditions for ASCs to arise in a non-pharmacological context, shedding light on the functional mechanisms of breathwork as well as its potential as a psychotherapeutic tool.

## Abstract (web summary)

Circular breathwork induces altered states of consciousness similar to psychedelics, predicted by decreases in CO2 saturation. Acute physiological and psychological dynamics during breathwork in turn predict sustained mental health benefits.

## Introduction

In recent years, growing evidence suggests that by evoking altered states of consciousness (ASCs) in a supportive setting, together with complementary processes such as heightened neuroplasticity, psychedelic-augmented therapy may alleviate some of the most wide-spread and debilitating forms of mental suffering, including post-traumatic stress disorder, depression and anxiety^1–6^. While these are hopeful developments, many patients who might benefit from such psychedelic treatments will not have access to them for the foreseeable future, whether due to legal restrictions, medical counterindications or financial limitations. A widely accessible treatment engaging similar therapeutic mechanisms could thus benefit a large population of patients struggling with mental health issues. In the current study, we explore the potential of circular breathwork to be such a complementary therapeutic tool. To this end, we track the acute physiological and experiential dynamics evoked by circular breathwork, as well as their impact on psychological well-being in the wake of a breathwork session.

Circular breathwork is a breathing technique rooted in traditional practices like Tummo and Pranayama Yoga, and subsequently adapted by a multitude of inter-related modern practices like Holotropic, Rebirthing, Conscious-Connected, Holorenic and Transformational Breathwork^7^. All these approaches have in common that over a prolonged period of time (approx. 15 min to several hours), participants sustain a deep, uninterrupted breathing rhythm, typically at a somewhat heightened speed. In addition, breathwork is typically conducted in a communal setting (i.e. in a group and/or in the presence of facilitators) and accompanied by emotionally evocative music. The term ‚circular breathwork‘ is thus used here as an umbrella term for a range of techniques in which inhale and exhale are actively connected to each other in a continuous cycle, maintaining an at least somewhat structured form of deliberate hyperventilation, which is embedded in a context of evocative music and social support. Table 1 highlights central common features—and differences—of the two circular breathwork techniques included in this study (Conscious-Connected and Holotropic breathwork).

**Table 1:** Commonalities and differences between the two breathwork practices examined in this study – Conscious-Connected breathwork and Holotropic breathwork

	Conscious-connected breathwork	Holotropic breathwork
Commonalities		
Circular breathing	Deeper and somewhat faster breathing, without pause between inhale and exhale	At session start, faster and slightly deeper breathing, connecting inhale and exhale into a continuous cycle. Participants are invited to find their own breathing rhythm once they feel they have entered their process.
Music		
(Loud & evocative at first, calmer towards the end)	✓	✓
Verbalization of experiences (‘sharing’) (before and after session)	✓	✓
Breathing position (typically lying down on a mat, potentially with a blindfold)	✓	✓
Differences		
Session duration	1–1.5 h	2.5–3 h (twice, see ‘Setting’)
Setting	Individual or group sessions; In group sessions, participants all take part in the session at the same time	Individual or group sessions; Group breathwork is conducted in pairs, so that one participant does breathwork (‘breather’) while the other observes supportively (‘sitter’). Pairs of participants exchange roles (‘breather’ and ‘sitter’) across two consecutive sessions.
Number of facilitators	Depending on style, 1 facilitator per 2–6 participants (i.e. ‘breathers’)	1 facilitator per up to 8 ‘breathers’
Breathing instructions	Potentially more specific: Participants are encouraged to take deep, full inhales and faster, relaxed exhales, connected to each other without pause.	Potentially more general: Participants are invited to become more aware of their breathing pattern, and then make it deeper and faster.
Facilitator support through physical touch (‘bodywork’)	Depending on facilitator style, potentially more proactive	Potentially more responsive (i.e. by request of breather) and less proactive
Creative expression interventions after breathwork (e.g. Mandala drawing)	–	✓

While anecdotal evidence from practitioners of circular breathwork suggests that it can benefit mental health, the first scientific investigations of these claims are only just emerging^8–10^—for an overview, see^7,11,12^. The studies conducted so far seem to generally demonstrate the benefits of circular breathwork for alleviating stress and anxiety^7,11–13^, reducing depression and PTSD^14^, and enhancing self-awareness and life satisfaction^7,10^. What’s more, these benefits have been hypothesized to be mediated by enhanced psychological openness^12,15^. This constellation of potential mental health benefits appears to closely resemble the one reported for psychedelics (e.g.^1–5^). Consistent with this, the subjective experiences arising acutely during circular breathwork have been tentatively aligned with those produced by psychedelic interventions—both anecdotally and in a small number of explorative studies^8^.

These observations open up several crucial questions regarding the mechanisms and effects of circular breathwork. First, to what extent do the immediate and sustained effects of breathwork resemble those of psychedelics? And if there are parallels to be drawn, what are the physiological and psychological mechanisms by which a reasonably simple shift in breathing rhythm can give rise to such effects, including altered states of consciousness?

Currently, insights into the physiological processes likely triggered by circular breathwork stem mainly (1) from medical studies of hyperventilation^16–20^, which often aim to model the physiological consequences of panic attacks, and (2) from studies examining the physiological consequences of the Wim Hof Method^21–23^ – a breathing technique that is related to circular breathwork but additionally features intermittent breath-holds and cold exposure. These studies suggest that circular breathwork increases blood oxygenation^16,23^ while reducing CO_2_ saturation^16–21,23^. This in turn renders blood pH more alkaline respiratory alkalosis,^16,18,19,21,23,24^, causing vasoconstriction^16,19,20,25–31^, particular throughout the neocortex^32–34^. However, it is not clear if and how these physiological changes play a causal role in altering participants‘ conscious experience, and if and how this in turn shapes psychological changes following a breathwork session. Alternatively, the subjective effects of circular breathwork may be largely independent of its physiological impact, relying instead more on psychological context factors like communal emotional expression, group sharing, evocative music, supportive touch by facilitators (‘body work’) and other contextual elements that breathwork practices are typically embedded in.

In this exploratory, mechanistic study we aimed to disentangle the contributions of these complementary mechanisms to the subjective experiences evoked by breathwork. To this end, we simultaneously tracked participants‘ end-tidal CO_2_ pressure (etCO_2_) and their subjective experiences throughout a breathwork session. We then explored if and how these processes were linked to each other, and if and how they predicted physiological and psychological changes following the session.

## Methods

### Participants and ethics

This exploratory, mechanistic study enroled 61 experienced breathwork practitioners via online advertisements distributed in various breathwork communities. Since we could only rely on a small pool of previous literature regarding the physiological and psychological effects of circular breathwork, we estimated the size of the recruited sample by considering psychedelic studies, which typically feature sample sizes of 15–25 participants^35^, and assuming that the effects of breathwork were likely to be at least somewhat less pronounced, bringing us to a conservative sample size of *n* = 32 per breathwork style. Due to participant drop-out on the day of the final scheduled sessions, this was when reduced to *n* = 30 and *n* = 31 for Holotropic and Conscious-Connected breathwork, respectively.

The average age of participants was 33.1 ± 7.1 years (Range: 20–55 years). 34 participants reported their gender as female, 22 male, 1 non-binary and 4 non-disclosed. Three participants reported being unemployed, eight reported being in education, the rest reported being self-employed (*n* = 17) or employed (*n* = 27). Information on race and ethnicity of participants was not collected. For an overview of participant demographics in each experimental group, see Supplementary Table S1.

The study was conducted at the premises of the MIND Foundation in Berlin between October 2021 and November 2022 and approved by the ethics committee of the Ärztekammer Berlin (File number Eth-55/21; Project Code PPEIA). The study was not preregistered. Written informed consent was given by all participants prior to the participation in the study after having an individual short interview with the study coordinator. Participants were included if they were between 18 and 60 years old, provided written informed consent and had previously experienced at least five immersive breathwork sessions. Exclusion criteria were: Presence of a mental health disorder in the forms of history of psychoses including schizophrenia, personality disorders (e.g. borderline or antisocial personality disorder) or a history of substance use disorder during the last 6 months (according to DSM-5). Cardiovascular diseases (e.g. inadequately treated arterial hypertension, heart arrhythmia), chronic lung disease (e.g. bronchial asthma) and chronic obstructive pulmonary disease, pregnancy, and the presence of a chronic disease of the central nervous system (especially epilepsy) were also excluded. At least one week before the session, potential participants filled out a questionnaire investigating all exclusion criteria. If they met all criteria for inclusion according to this questionnaire, participants next conducted a ‘check-in interview’ per phone call with the study coordinator, where they were informed about the structure of the study (including session length, explanation of CO_2_ measurements, and online questionnaires pre- and post-session where applicable). During this phone interview, participants were also asked once more about potential exclusion criteria to make sure e.g. relevant diagnoses had not been overlooked. Finally, participants received their personal pseudonymized code, which they used both in the session itself but also for the online questionnaires pre- and post-session. Participants were not compensated financially for their study participation.

### Interventions: holotropic and conscious-connected breathwork

The current study explored the effects of two popular forms of circular breathwork: Holotropic breathwork^36,37^ and Conscious-Connected breathwork^38^. To make sure that participants engaged in the breathwork style that felt most comfortable to them, participants could freely choose whether to take part in a Holotropic (*N* = 30) or Conscious-Connected (*N* = 31) breathwork session. In addition, to disentangle the effects of the breathing technique itself from effects of the broader session setting, 18 of 61 participants were randomly assigned to a control (passive-breath) group, in which they were instructed to participate in all aspects of the breathwork session but adhere to their every-day breathing rhythm (see below).

Altogether, we conducted two Holotropic and three Conscious-Connected sessions. On the day of each session, every participant checked in with the study coordinator, completed a COVID-test, and underwent a short medical examination to check for physiological exclusion criteria (resting heart rate > 100 beats per minute, resting blood pressure > 160, obvious issues in lung function that could be detected by a medical doctor using a stethoscope). Then participants got handed a randomly selected envelope in a separate room by the study coordinator—assigning them either to the control (passive breath) or the active breath condition (which both took part in the same breathing session). For participants assigned to the active-breath condition, the study coordinator then explained that they should follow all breathing instructions by the facilitators. For participants assigned to the control (i.e. passive-breath) condition, the study coordinator instructed that they should continue their normal breathing rhythm, ignoring breathing instructions by the facilitators. Participants assigned to the control and active-breath conditions took part in the same breathwork session to equalize context factors like music, duration, pre-session sharing and social contagion across participants as much as possible between the two conditions. Additionally, all participants were asked to not reveal their group assignment to anybody before the end of the session, in order to blind the facilitators as much as possible. One should note however that facilitators may nevertheless have deduced group assignments based on the participants’ breathing rhythm.

To disentangle the effects of the breathing technique from effects of the broader session setting, participants were randomly assigned to the active- or passive-breath groups at a ratio of approximately 70:30, so that ultimately 43 out of 61 participants (20 out of 30 in Holotropic breathwork sessions, and 23 out of 31 in Conscious-Connected breathwork) were in the active-breath condition, where they changed their breathing rhythm according to the respective breathwork format they had chosen, while 18 participants (10 for Holotropic, and 8 for Conscious-Connected) were in the passive-breath condition, adhering to their normal breathing rhythm.

Sessions comprised 7-15 participants and were supported by experienced breathwork facilitators trained in the respective breathwork technique. Facilitators were only briefed that there were some participants that had been asked to adhere to their normal breathing pattern, but were not informed how many and which participants were assigned to this experimental condition. In both formats, a group sharing was conducted before the breathwork session, where participants were invited to express thoughts and feelings about their current state, or set an intention for the session. Another sharing round was done following the session to integrate the experience. Also in both formats, facilitators could offer physical support (e.g. a hand on the shoulder) and bodywork (e.g. letting participants push against the hands of the facilitator) when participants were encountering intense emotional experiences. Throughout the session, 2–3 study helpers assisted with measurements of etCO_2_ and experience depth, while interfering as little as possible in the participants’ experiences.

For the **Holotropic breathwork** sessions, the day consisted of 2 consecutive breathing sessions that each lasted about 3 h and which participants experienced together in pairs, such that half of the group would breathe first, and the other half would play the role of sitters who were there to provide emotional and practical support (e.g. by providing water or a blanket), irrespective whether they had been assigned as active or passive breathing participant. Sitters were not counted as part of the passive-breath group – in fact, data from sitter sessions were not analysed at all. In the second breathing session, each pair of participants reversed roles, such that sitters from the first session would be breathers (assigned either to the passive or active breath group) in the second session, and vice versa. Each session was supported by 2–4 facilitators who gave the breathing instruction to ‘breathe somewhat more deeply and intensely than usual’. Right after the session, participants were invited to engage in mandala drawing (a form of graphic expression of the experience where participants get a white paper with a round shape drawn on it and fill that with colours). This type of drawing is traditionally used as a form of non-verbal integration of the breathwork experience in Holotropic breathwork settings.

For the **Conscious-Connected breathwork** session, one breathwork session lasted about 1,5 h and was conducted jointly for all participants at the same time (there were no sitters, so the group consisted of active and passive breathers only). Sessions were accompanied by two facilitators per group who gave about the following breathing instructions: “Breathe a little bit deeper and more intensely than usual into the belly, and connect your breaths into a circle without a pause between in- and exhale.”

### End-tidal CO_2_ pressure

End-tidal partial CO_2_ pressure (etCO_2_; i.e. partial pressure of CO_2_ at the end of exhalation) was measured at 6 time points throughout the breathwork session using a portable CO_2_-breathalyser (capnography) device (EMMA, produced by Masimo, Neuchatel, Switzerland). Since Holotropic breathwork sessions lasted up to three hours, this equated to a measurement of etCO2 every 25–30 min; while Conscious-Connected breathwork sessions lasted approx. 1.5 h, equating to a measurement every 15–20 min. Before the session, participants were instructed how to use these capnography devices. At each measurement time point, the study helpers would alert participants with a touch on the shoulder, then put the device in the participant’s hand, and together lead their hand to their mouth, where the participants would inhale freely and then exhale as fully as possible through a tube attached to the device. The breathalyser then displayed both the wave-form of CO_2_ pressure throughout the exhalation (capnographic display), and the etCO_2_, which was noted down by the study helpers.

### Subjective experience

To cause minimal disruption to the ongoing session, experiential depth was assessed using hand signs to provide a depth rating from 1 to 5 at the same six time points as etCO_2_ throughout the session. One finger raised indicated normal waking consciousness, while five fingers (or inability to raise one’s hand) signalled deep altered states of consciousness. Two to four raised fingers indicated intermediate states (see below). The hand signs were demonstrated and explained to all participants before the beginning of the session. More detailed examples of criteria for the subjective depth ratings, which were also verbally shared with the participants before the start of the session were as follows:

1. Normal ability to speak, normal analytical thinking, clear awareness of time, space and current context, normal experience of self, including awareness of biographical data, social context, and conscious control over actions.
2. Mostly normal thinking and speaking ability, mild changes e.g. in perception, emotional experience of spontaneous body movements.
3. Reduced analytical thinking, partial loss of awareness over time and space, reduced awareness of self, potentially some non-immersive visual or auditory hallucinations, emotional experiences of involuntary body movements.
4. Loss of analytical thinking, strongly reduced awareness of self, time and space, reduced reaction to external stimuli, potentially visual or auditory hallucinations, uncontrolled emotional expression, or body movements.
5. Loss of awareness of self, time, and space, strongly reduced perception of external stimuli, strongly reduced ability to speak or control physical movements, potentially intense and immersive visual or auditory hallucinations, uncontrolled emotional expression, or body movements.

### Sub-acute self-reports on subjective experience

Next, we set out to quantify the subjective experiences encountered by participants throughout the session in more detail. As a first benchmark, we administered two post-hoc surveys of altered states of consciousness that are regularly used to quantify psychedelic experiences: The 11-Dimensional Altered States of Consciousness Scale (11D-ASC^39^), and in a subgroup of 33 participants also the Mystical Experiences Questionnaire 30 (MEQ30^40,41^). Both questionnaires were administered within one hour of the session. Subjective experience of study participants was assessed immediately after the breathwork session using widely established scales for measuring altered states of consciousness: the 11-Dimensional Altered States of Consciousness Scale (11-DASC^39^) and Mystical Experiences Questionnaire (MEQ30^40,41^). 11-DASC subscales were computed as: “Oceanic boundlessness” (experience of unity, spiritual experience, blissful state, insightfulness), “Ego dissolution” (disembodiment, impaired control and cognition, anxiety) and “Visual reconstruction” (complex imagery, elementary imagery, audio-visual synaesthesia, changed meaning of perception). The questionnaires were handed out on paper and took around 20–25 min to complete.

### Self-reports on mental health and well-being

To explore follow-on effects of breathwork on mental health and wellbeing, the first half of the participants (33 out of 61) were asked to fill out self-report questionnaires one week before and one week after the breathwork session (these metrics could not be recorded for the second half of participants for logistical reasons). The self-report version of the 16-item Quick Inventory of Depressive Symptomatology (QIDS-SR16)^42^ was used to assess depressive symptoms and the Warwick-Edinburgh Mental Wellbeing Scale (WEMBWS)^43^ was used to measure overall wellbeing. These questionnaires were administered as online surveys using a secure platform (implemented by Gravity Forms), hosted on the MIND Foundation’s website. Entries were pseudonymized, i.e. participants identified with their pseudonymized study code only. The questionnaires took around 10 min in total to fill out.

To ensure that follow-on metrics mainly reflected the effects of the breathwork session rather than events or activities that might have taken place in the meantime, we also asked participants to list any emotionally significant events and/or self-development practices that had taken place between the breathwork session and the time of filling out the follow-up questionnaires. According to the responses to this question, two participants engaged in daily meditation practices following the breathwork session, two started new romantic relationships, and one had a psychotherapy session prior to completing the one-week follow-up survey. We assume that these activities did not significantly impact outcome scores.

Out of the 33 participants that were given self-report questionnaires, 8 failed to fill out the questionnaires on at least one of the two measurement time points. Given such missing values, we chose to only include complete sets of responses in our analysis. As such, the analysis ultimately included the responses of 25 participants – 20 in the active-breath and 5 in the passive-breath condition (see Supplementary Table S2).

### Measurement of α-amylase and IL-1β in saliva

To establish whether breathwork also caused physiological changes, particularly in terms of activity in the autonomic nervous system (ANS), we tracked concentrations of two molecular markers in saliva, extracted directly before and after the breathwork session: The inflammatory marker interleukin-1 beta (IL-1β), and alpha-amylase (α-amylase), which is a proxy for ANS activity, particularly its sympathetic branch^44,45^. Levels of salivary α-amylase and IL-1β were assessed before and after the breathwork session. Prior to session participants were instructed how to use saliva collection tubes Salivette, Sarstedt Germany. After sample collection, tubes were frozen and kept at –20 C until analysis using enzyme-linked immunosorbent assay (ELISA). On the day of assays, saliva samples were thawed completely, vortexed and centrifuged at 1500 × *g* for 15 min to remove mucins that precipitate during freezing. Samples and assay kits (salivary α-amylase assay kit cat no. 1-1902; salivary IL-1β ELISA kit cat no. 1-3902) were brought to room temperature before use and assays were performed following manufacturer’s instruction (Salimetrics). All samples were run in duplicates. Plates were read using Clario Star plate reader (BMG Labtech) and final α-amylase and IL-1β concentrations calculated according to protocol´s instructions using MARS data analysis software. Natural logarithm transformation^46^ was applied to biomarkers concentration and those values were then used in statistical testing. 6 of the samples we gathered from 61 participants yielded inconclusive results, likely due to marker concentrations remaining below detection range. As such, our biomarker analyses ultimately included measurements from 55 participants.

### Data analysis

Questionnaires that had been administered on paper were subsequently transcribed by hand into Excel tables, and all transcriptions were checked independently by at least one other person. Data were then analysed using custom-written code in MATLAB and R (Version 4.2.3). For average scores of subjective experience depth and/or etCO_2_ per participant, as shown e.g. in Fig. 3A, B, we averaged the scores for measurement time points 2–4, i.e. from the most active part of the session. Note that while measurements of etCO_2_ and subjective experience throughout the session were recorded for all 61 participants, both the subsequent self-reports of mental well-being and the biomarker measurements from saliva encountered some missing measurements (see above). Participants were only included in an analysis when a full data set for that analysis was available.

### Statistics

Statistical tests were conducted using standard functions (e.g. ANOVA, t test and ttest2) in MATLAB and R (Version 4.2.3). Significant results were reported at *p* < 0.05, and all tests were applied two-sided. Throughout the study, we employed the following statistical tests.

1. Group differences between active and passive breath groups as well as across breathwork techniques in terms of mean/min etCO_2_ and mean/max experience depth ratings:
  Two-way ANOVA with interaction term, with active/passive breath and breathing technique as predictors. Average experience depth did not significantly deviate from normal distribution (Shapiro-Wilk test; W = 0.97, *p* = 0.19), while maximum experience depth did (W = 0.88; *p* < 0.001). Both average etCO2 levels and minimum etCO2levels per participant also significantly deviated from normal distribution (respectively W = 0.95, *p* = 0.009 and W = 0.95; *p* = 0.02). Note however that even for statistically significant departures from normality, the W metric (range from 0 to 1, with 1 signalling perfect adherence to a normal distribution) was still very high at a range from 0.88 to 0.95. As such, we decided that deviations from normality were small enough that ANOVAs could be computed robustly. Equality of variances was not formally tested.
2. Differences in 11-DASC and MEQ scores from reference scores, either for psilocybin, LSD, MDMA or placebo treatment (see data base analysis below):
  Individual one-group t tests for differences from reference scores per questionnaire sub-scale; with Dunn-Sidak correction for multiple comparisons across sub-scales. These *t* tests were conducted independently for active and passive breath groups. MEQ scores did not significantly deviate from normal distribution (Shapiro-Wilk test; W = 0.960, *p* = 0.31). For the 11-DASC, 8 of 11 sub-scales conformed to the normality assumption (W = 0.96 to 0.98; *p* = 0.12 to 0.68). The remaining 3 sub-scales deviated statistically significantly (*p* < 0.001 to 0.04), but at high W values (W = 0.90 to 0.95). Equality of variances was not formally tested. Bayes Factors (bf01) were computed to test the likelihood that the null hypothesis (lack of difference between breathwork scores and reference scores) held, which would be indicated by a bf01 > 1. bf01 values were computed by applying the bf.t test function, which is part of the open-source BayesFactor MATLAB package developed by Bart Krekelberg^47^.
3. Differences in 11-DASC and MEQ scores between active and passive breath groups:
  Two-way ANOVA with interaction term, with active/passive breath and questionnaire sub-scale as predictors. Equality of variances was not formally tested. For potential deviations from normal distribution as well as the computation of bf01, see point 2.
4. Pre-post measurements of QIDS-SR16 scores, WEMBWS scores and α-amylase and IL-1β levels:
  Paired t tests of pre- versus post-values. *T* tests were conducted separately for passive and active breath groups. Differences from normal distribution were not statistically significant for QIDS scores (pre-breathwork: W = 0.96, *p* = 0.28; post-breathwork: W = 0.97, *p* = 0.72) and WEMBWS scores (pre: W = 0.97, *p* = 0.47; post: W = 0.93, *p* = 0.07). Deviations of log-normalized α-amylase and IL-1β levels were also small. Log-normalized IL-1β measurements did not significantly deviate from normal distribution (IL-1β pre: W = 0.96, *p* = 0.07; IL-1β post: W = 0.98, *p* = 0.59). Equality of variances was not formally tested. Log-normalized α-amylase measurements deviated significantly from normal distribution pre-session (W = 0.93; *p* = 0.004) though at a high W value, indicating practically negligible deviations, post-session α-amylase measurements did not deviate significantly from normality (W = 0.98; *p* = 0.44). Equality of variances was not formally tested.
5. For all *t* tests and ANOVAs, Cohen’s D was computed as a measure of effect size^48–50^, together with 95% confidence intervals based on the standard error^48,51,52^. For paired t tests, Cohen’s D was computed as Cohen’s D_4_^48^ based on the correlation between paired measurements:
  $${D}_{4}=\frac{{mean}({post}-{pre})}{{stdev}\left({post}-{pre}\right)\times \sqrt{1-{r}_{{pre},{post}}}}$$
  where pre and post are series of paired measurements (e.g. QIDS scores before and after breathwork intervention), and r is the correlation between the paired measurements. See Supplementary Table S3 for details on all statistical metrics, custom code provided on (https://github.com/zero-noise-lab/BreathworkCO2).
6. All correlations were computed as non-parametric Spearman correlations, with 95% confidence intervals provided by the MATLAB function ‘corrcoef’.
7. For analysing the relationship between subjective experience depth and etCO_2_ measured at multiple time points we performed multilevel modelling (linear mixed-effects model) using lme4 package in R. Experience depth was modelled as outcome variable and etCO_2_ and time as predictor variables separately for active and passive-breath groups.

### Database analysis of MEQ30 and 11-DASC reference scores

To estimate reference scores of typical MEQ30 and 11-DASC outcomes for psychedelic versus placebo treatments, we relied on data reported in the Altered States Database (ASDB; ; see Prugger et al., 2022). To estimate reference scores for ASCs triggered by psilocybin, LSD and MDMA, we searched the database for studies in which participants had received a standard therapeutic dose of each substance (approx. 20–25 mg of psilocybin at an adult body weight of 75-80 kg; 125 mg of MDMA; 100 microgram of LSD), and subsequently completed the MEQ30 and/or 11-DASC. To gather reference scores for MEQ30 and 11-DASC after a placebo treatment, we included studies that had administered psychopharmacologically neutral liquids as a placebo treatment (water, saline, saline with glucose, lactose, and in one case a very low concentration of alcohol solution). The studies included as a result of this search strategy are listed in Supplementary Tables S4, S5. For each questionnaire sub-scale, reference scores were then computed as a weighted average of mean questionnaire score per study, weighted by the number of participants reported for each study in the ASDB, as can be seen here:

1 $$S=\frac{{\sum }_{1}^{n}{s}_{n}\times {p}_{n}}{{\sum }_{1}^{n}{p}_{n}}$$

where S is the overall score in a questionnaire sub-scale, n is the number of included studies, s_n_ is the average score in each study, and p_n_ is the number of participants in each study.

### Reporting summary

Further information on research design is available in the Nature Portfolio Reporting Summary linked to this article.

## Results

The current study explores the effects of two popular forms of circular breathwork: Holotropic breathwork and Conscious-Connected breathwork. To this end, we tracked end-tidal partial CO_2_ pressure (etCO_2_) and subjective experience depth at six time points throughout a breathwork session in 61 experienced breathwork practitioners. Since Holotropic breathwork sessions lasted up to three hours, this equated to a measurement every 25–30 min while Conscious-Connected breathwork sessions lasted approx. 1.5 h, equating to a measurement every 15–20 min. To emulate the typical features of breathwork as it is currently practiced, sessions were held in groups of 7-15 participants and included a group sharing before and after breathwork, as well as evocative music and support by trained facilitators throughout the session (see Methods). To disentangle the effects of these broader setting features from those of the breathing technique itself, in each session we asked approx. 70% of participants to actively engage in circular breathing (active-breath condition), while the remaining participants were instructed to adhere to their normal breathing rhythm while participating in the same session (passive-breath condition; see Methods).

### End-tidal CO_2_ saturation during breathwork

**Fig. 1:**
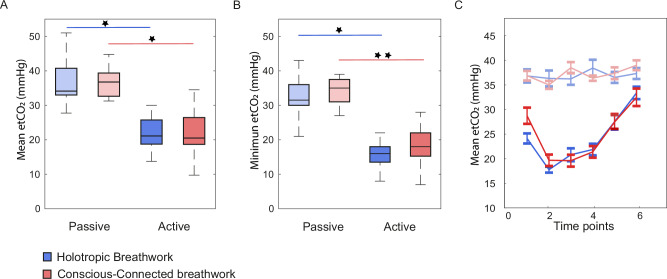
End-tidal CO_2_ saturation during breathwork. **A** Boxplot of mean partial end-tidal CO_2_ pressure (etCO_2_) per participant, pooled across the active portion of the breathwork session (time points 2–4 out of 6). Central line: Median. Box outline: 25th and 75th percentile. Whiskers: 10th and 90th percentile. Blue: Holotropic (*n* = 20 participants for active-breath group, *n* = 10 for passive-breath group). Red: Conscious-Connected (*n* = 23 participants for active-breath group, *n* = 8 for passive-breath group). Desaturated colours (on the left): Passive-breath control groups. Saturated colours (on the right): Active-breath groups. Both active-breath groups showed significantly lower etCO_2_ than their passive-breath counterparts. **B** Same as A for minimum etCO_2_ per participant across measurement time points 2–4. Differences between active and passive breath groups are even more pronounced, with active-breath participants reaching etCO_2_ as low as 10–20 mmHg throughout the session. **C** Time course of etCO_2_ across all six measurement time points. Error bars: SEM. Blue: Holotropic (*n* = 20 participants for active-breath group, *n* = 10 for passive-breath group). Red: Conscious-Connected (*n* = 23 participants for active-breath group, *n* = 8 for passive-breath group). etCO_2_ decreased rapidly at the start of the session, reaching their minimum around measurement time points 2–3, and then increasing gradually towards the end of the session. Note that for Conscious-Connected breathwork sessions, measurements were taken approx. every 15 min, while for Holotropic breathwork sessions, measurements were taken approx. every 30 min (see “Methods” and Table 1).

To first establish if circular breathwork caused CO_2_ saturation to drop as previously described for voluntary hyperventilation^16,18,19,53^, we repeatedly measured etCO_2_ throughout the session (see Methods). As expected, etCO_2_ in active breathers dropped significantly throughout the session compared to passive breathers, with an average of *36.7* ± *1.5* versus *20.1* ± *0.8* mmHg (*mean* *±* *SEM*; see Fig. 1A; for individual etCO_2_ traces, see Supplementary Fig. S1). This effect occurred irrespective of Holotropic or Conscious-Connected breathing techniques (*Two-way ANOVA; df* = *60; Effect of active versus passive breathing: F* = *111.4; p* < *0.001; Cohen’s D: 3.03; Effect of breathing technique: F* = *0.0; p* = *0.91; Cohen’s D: 0.14; Interaction: F* = *0.0; p* = *0.91; see* Supplementary Table S3*for details*). Effects were even more pronounced for the minimum etCO_2_ per participant across time points 2–4, which dropped to *16.6* ± *0.8* *mmHg* in the active-breathing group but remained at *34.3* ± *1.5* *mmHg* in the passive-breathing group (*mean* *±* *SEM;* Fig. 1B*; Two-way ANOVA; df* = *60; Active versus passive breathing: F* = *119.0; p* < *0.001; Cohen’s D: 3.11; Breathing technique: F* = *0.1; p* = *0.81; Cohen’s D: 0.06; Interaction: F* = *0.2; p* = *0.64; see* Supplementary Table S3*for details*). The time course of etCO_2_ confirmed that in active-breathing sessions, etCO_2_ levels dropped throughout the first half of the session, typically reaching their minimum at measurement time points 2–3, and then gradually rising again towards the end of the session (Fig. 1C). These dynamics were highly similar between Holotropic and Conscious-Connected breathwork sessions (*Correlation of average CO*_*2*_ *trajectories for active-breath groups in Holotropic and Conscious-Connected breathwork: n* = *6 observation time points per breathwork style; r* = *0.92; conf.int(r)*_*low*_ = *0.41; conf.int(r)*_*high*_ = *0.99; p* = *0.01*). These results indicate that participants adhered to the breathing instructions they were assigned to, with the active-breath group, but not the passive-breath group, reaching decreases in etCO_2_ that would be expected during deliberate hyperventilation.

### Subjective experience depth during breathwork

**Fig. 2:**
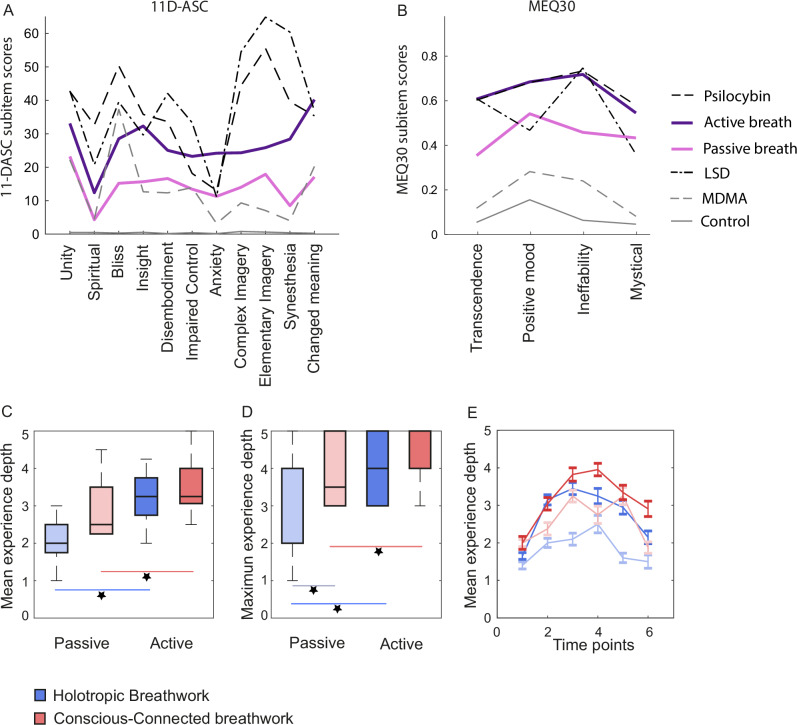
Experience depth during breathwork. **A** Subjective experience triggered by breathwork, as assessed by the 11-Dimensional Altered States of Consciousness Scale (11D-ASC). Dark purple: Active-breath condition (*n* = 43 participants). Light purple: Passive-breath condition (*n* = 18 participants). Dashed lines: Typical 11D-ASC scores for three typical psychedelic treatments (see in-figure legend), with data extracted from the Altered States of Consciousness Database (see “Methods” and Supplementary Table S4): 20–25 mg Psilocybin (oral; scores pooled across five relevant clinical studies; see Supplementary Table S4), 0.1 mg LSD oral/0.075 mg intravenous (pooled across six studies), and 0.125 mg MDMA (oral; pooled across five studies) .Solid grey line: Typical 11D-ASC scores for placebo treatments (three clinical studies, see Supplementary Table S4). **B** Same as (**A**) for the four subscales of the Mystical Experiences Questionnaire 30 (MEQ30). Reference studies for psychedelic and placebo treatments are listed in Supplementary Table S5. **C** Boxplot of mean experience depth per participant, determined by hand signs given on a scale of 1–5 (1= every-day consciousness, 5 = deeply altered consciousness; see “Methods”), which were then pooled across measurement time points 2–4. Central line: Median. Box outline: 25th and 75th percentile. Whiskers: 10th and 90th percentile. Colour scheme as in (**A**, **B**). Holotropic: *n* = 20 participants for active-breath, *n* = 10 for passive-breath. Conscious-Connected: *n* = 23 participants for active-breath, *n* = 8 for passive-breath. Active-breath groups on average indicated deeper experiences than their passive-breath counter parts. **D** Same as (**C**) for the maximum experience depth. **E** Same time course as shown in Fig. 1C, but for experience depth. Holotropic: *n* = 20 participants for active-breath, *n* = 10 for passive-breath. Conscious-Connected: *n* = 23 participants for active-breath, *n* = 8 for passive-breath. In both active-breath groups, consciousness is increasingly altered at the start of the session, reaching its maximum around measurement time points 3–4, and then gradually reverts to baseline. A qualitatively similar but quantitatively shallower dynamic can be seen in both passive-breath groups. Note that for Conscious-Connected breathwork sessions measurements were taken approx. every 15 min, while for Holotropic breathwork sessions, measurements were taken approx. every 30 min (see “Methods” and Table 1).

Next, we quantified the subjective experiences encountered by participants throughout the session. As a first benchmark, we administered two surveys of altered states of consciousness classically used to quantify psychedelic experiences: The 11-Dimensional Altered States of Consciousness Scale (11D-ASC^39^), and in a subgroup of 33 participants also the Mystical Experiences Questionnaire 30 (MEQ30^40,41^). Our results show that the subjective experiences reported by participants resemble those triggered by psychedelics across several experiential qualities (Fig. 2A, B; reference data sets extracted from^35^). Specifically, the 11-DASC and MEQ30 scores reached by active breathers were significantly higher than scores that would be expected for a placebo treatment (see “Methods” for composition of reference data set; *t tests for difference from placebo reference scores; 11-DASC: df* = *42; t* = *6.4 to 13.9 across 11 sub-scales; Cohen’s D* = *0.97 to 2.11; all p* < *0.001; all differences significant at a family-wise error rate of 0.05, based on Dunn-Sidak correction for multiple comparisons; MEQ30: df* = *21; t* = *10.2 to 12.9 across 4 sub-scales; Cohen’s D* = *2.17 to 2.98; all p* < *0.001; all differences significant at a family-wise error rate of 0.05 based on Dunn-Sidak correction; see* Supplementary Table S3). They also approached scores evoked e.g. by a commonly used moderate therapeutic dose of 20–25 mg of psilocybin, with scores on all four sub-scales of the MEQ-30 and 2 of 11 sub-scales of the 11-DASC being statistically indistinguishable between breathwork and psilocybin based on a post-hoc Bayes Factor analysis, and one sub-scale of the 11-DASC in fact scoring higher for breathwork than psilocybin (see Methods for composition of reference data; *t tests for difference from psilocybin reference scores with subsequent Bayes Factor analysis; 11D-ASC: df* = *42; t* = *-10.8 to 5.6 across 11 sub-scales; Cohen’s D* = *0.18 to 1.65; all p* < *0.001 to 0.12; scores significantly lower than psilocybin scores in 6 of 11 sub-scales at a family-wise error rate of 0.05 based on Dunn-Sidak correction; Bayes Factors: bf01* < *0.001 to 3.26; bf01* > *1 in 2 of 11 scales, indicating likelihood of H*_*0*_ *; MEQ30: df* = *21; t* = *-0.7 to 0.1 across 4 sub-scales; Cohen’s D* = *0.02 to 0.15; p* = *0.50 to 0.94; all differences insignificant at a family-wise error rate of 0.05 based on Dunn-Sidak correction; Bayes Factors: bf01* = *3.8 to 4.7; bf01* > *1 in all 4 sub-scales;* see Supplementary Table S3). Similar, though less consistent, results were obtained when comparing breathwork to a typical therapeutic dose of 0.1 mg LSD (*t tests for difference from LSD reference scores; 11D-ASC: df* = *42; t* = *0.9 to 13.9 across 11 sub-scales; Cohen’s D* = *0.14 to 2.12; all p* < *0.001 to 0.37; breathwork scores significantly lower than LSD scores in 9 of 11 sub-scales at a family-wise error rate of 0.05; bf01* < *0.001 to 4.16; bf01* > *1 for 2 of 11 sub-scales; MEQ30: df* = *21; t* = *0.0 to 4.9 across 4 sub-scales; Cohen’s D* = *0.01 to 0.80; p* < *0.001 to 0.97; ; breathwork scores significantly lower than LSD scores in 2 of 4 sub-scales at a family-wise error rate of 0.05; bf01* < *0.001 to 4.7; bf01* > *1 in 2 of 4 MEQ sub-scales; see* Supplementary Table S3), and in comparison to MDMA, the reported subjective effects of breathwork were in fact stronger across a majority of questionnaire sub-scales (*t tests for difference from MDMA reference scores; 11D-ASC: df* = *42; t* = *4.0 to 10.6 across 11 sub-scales; Cohen’s D* = *0.62 to 1.62; all p* < *0.001; breathwork scores significantly higher than MDMA scores in all 11 sub-scales at a family-wise error rate of 0.05; MEQ30: df* = *21; t* = *9.2 to 11.5 across 4 sub-scales; Cohen’s D* = *1.96 to 2.44; p* < *0.001 for all sub-scales; ; breathwork scores significantly higher than MDMA scores in all 4 sub-scales at a family-wise error rate of 0.05; see* Supplementary Table S3)

For passive breathers, subjective effects were significantly smaller than for active breathers (Two-way ANOVA; 11-DASC: df = 670; passive versus active breath: F = 94.0; Cohen’s D = 0.77; *p* < 0.001; Sub-scales: F = 6.4; Cohen’s D = 0.15 to 1.49 across pair-wise sub-scale comparisons; *p* < 0.001; Interaction: F = 1.4; *p* = 0.19; MEQ30: df = 119; passive versus active breath: F = 19.0; Cohen’s D = 0.22; p < 0.001; Sub-scales: F = 2.4; Cohen’s D = 0.11 to 0.60 across pairwise comparisons between sub-scales; *p* = 0.07; Interaction: F = 0.7; *p* = 0.53; see Supplementary Table S3). However, scores for the passive-breath group still differed noticeably from typical placebo scores (*t* tests for difference from placebo scores; 11-DASC: df = 17; t = 2.5–7.3 across 11 sub-scales; Cohen’s D = 0.59–1.72; *p* < 0.001–0.02; differences significant at a family-wise error rate of 0.05 in 10 of 11 sub-scales based on Dunn-Sidak correction; MEQ30: df = 7; t = 4.1–5.6 across 4 sub-scales; Cohen’s D = 1.44–1.99; all *p* < 0.01; differences significant at a family-wise error rate of 0.05 in all 4 sub-scales; see Supplementary Table S3).

To further explore the dynamics of subjective breathwork experiences over the course of the session, we also asked participants to rate the depth of their subjective experience at the same six time points at which etCO_2_ was measured throughout the session via simple hand signs: One raised finger signified ordinary waking consciousness, while five raised fingers signified deeply altered consciousness, and 2–4 raised fingers correspondingly signified intermediate states (for details, see “Methods”). To test if hand-sign scores throughout the session accurately reflected subjective experience depth, we correlated the average depth ratings across measurement time points 2–4 to the 11-DASC and MEQ30 scores which participants indicated post-hoc (see Fig. 2A, B). All eleven sub-scales of the 11-DASC correlated positively with hand-sign ratings, as did all four sub-scales of the MEQ30 and the total MEQ30 score (see Supplementary Fig. S2). This suggests that hand signs were a valid metric of subjective experience at any given moment.

The ratings given by hand sign throughout the breathwork session indicated profoundly changed consciousness. The average depth of experience ratings across measurement time points 2–4 were clearly higher in active than passive breathers (*mean* *±* *SEM: 3.46* ± *0.11 versus 2.46* ± *0.20 ; Two-way ANOVA; df* = *60; Effect of active versus passive breathing: F* = *20.8; Cohen’s D* = *1.31; p* < *0.001; see* Supplementary Table S3*)*, and somewhat higher in Conscious-Connected than Holotropic breathwork sessions (Fig. 2C*; mean* *±* *SEM: 3.40* ± *0.16 versus 2.92* ± *0.15; Two-way ANOVA; df* = *60; Effect of breathing technique: F* = *4.8; Cohen’s D* = *0.56; p* = *0.03; Interaction : F* = *0.4; p* = *0.53*). This pattern remained clearly present when considering maximum rather than average experience depths across measurement time points 2–4 (Fig. 1D), both for the difference between active and passive breathing (*mean* *±* *SEM: 4.12* ± *0.12 versus 3.22* ± *0.30; Two-way ANOVA; df* = *60; Active versus passive breath: F* = *15.0; Cohen’s D* = *1.13; p* < *0.001; see* Supplementary Table S3) and between breathwork techniques (*mean* *±* *SEM: 3.88* ± *0.35 versus 2.70* ± *0.40; Two-way ANOVA; df* = *60; Conscious-Connected versus Holotropic: F* = *5.1; Cohen’s D* = *0.54; p* = *0.03; Interaction: F* = *1.0; p* = *0.33*). The difference in depth ratings between breathwork techniques appeared to be particularly driven by the fact that the passive-breath group rated their experience as deeper in Conscious-Connected than in Holotropic breathwork sessions *(mean* *±* *SEM: 2.90* ± *0.30 versus 2.05* ± *0.18; post-hoc t test: df* = *15; t* = *2.41; Cohen’s D* = *0.69; p* = *0.03*).

In terms of temporal dynamics throughout the session, the most intense subjective experiences were likely to occur towards the middle of the session, ramping up during time points 1–2, and waning again through time points 5 and 6 (Fig. 2E). These dynamics closely follow those of the etCO_2_ measurements shown in Fig. 1C. Interestingly, they also applied almost identically to holotropic and conscious-connected breathwork (Fig. 2E; *Correlation of average depth trajectories for active-breath groups in Holotropic and Conscious-Connected breathwork: n* = *6 observation time points per breathwork style; r* = *0.90; conf.int(r)*_*low*_ = *0.35; conf.int(r)*_*high*_ = *0.99; p* = *0.01*), despite the fact that the absolute duration of a Holotropic breathwork session was almost double that of a Conscious-Connected session (approx. 3 versus 1.5 h).

### Relationship between end-tidal CO_2_ and experience depth

**Fig. 3:**
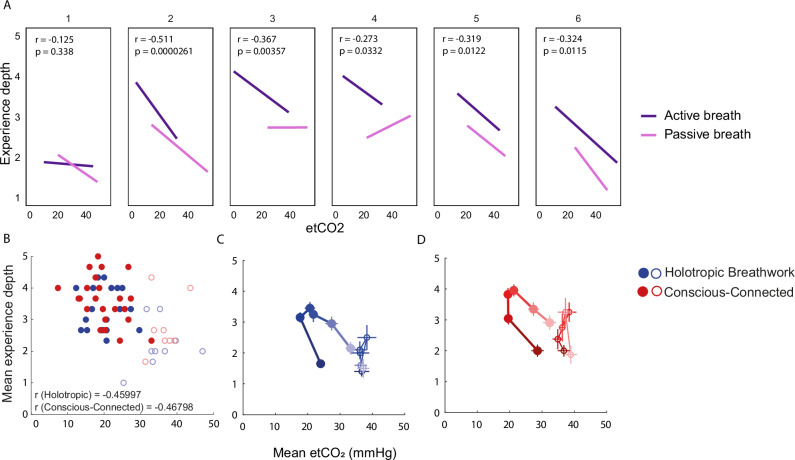
Relationship between end-tidal CO_2_ saturation and experience depth. **A** Regression between etCO_2_ and experience depth, separated into six measurement time points. Dark purple: Active-breath condition (*n* = 43 participants). Light purple: Passive-breath condition (*n* = 18 participants). **B** Scatter plot showing mean experience depth per participant, averaged across measurement time points 2–4, as a function of mean end-tidal CO_2_ pressure (etCO_2_) averaged across the same interval (Holotropic: *n* = 30 participants; Conscious-Connected: *n* = 31 participants). **C** Average trajectory of etCO_2_ and experience depth throughout a session of Holotropic breathwork. Filled circles: Average etCO_2_ and experience depth at each time point, averaged across participants of the active-breath group. Thin horizontal and vertical lines: SEM for etCO_2_ and experience depth, respectively, for each time point (*n* = 30 participants). Darker colours signify measurement time points at the beginning of the session, light colours denote time points towards the end of the session. Open circles: Same for the passive-breath control group. **D** Same as (**B**) for Conscious-Connected breathwork (*n* = 31 participants).

To establish whether physiological and experiential dynamics during the session related to each other in a way that might suggest a causal relationship, we first correlated average etCO_2_ values in the active part of the session (time points 2–4) with average experience depth for the same time points. As shown in Fig. 3A, B, there was a considerable correlation (*Holotropic: r* = *–0.46; conf.int(r)*_*low*_ = *–0.70; conf.int(r)*_*high*_ = *-0.12; df* = *29; p* = *0.012; Conscious-Connected: r* = *–0.47; conf.int(r)*_*low*_ = *–0.71; conf.int(r)*_*high*_ = *–0.15;df* = *30; p* = *0.008*). This indicated that participants who reached lower etCO_2_ levels throughout the session were also more likely to experience deeper ASCs. To explore if this relation held on a moment-by-moment basis, we also quantified the relationship between the raw etCO_2_ measurements and experience depth across all six measurement time points in 61 participants, using a multivariate mixed-model analysis. More specifically, we tested to what extent experience depth was predicted by etCO_2_ levels and time. Our analysis indicated that experience depth was supported both by decreases in etCO_2_ and by advancing time in the session in the active-breath condition (*Active breath: estimated fixed effects coefficient for etCO2* = *–0.61; Std.Error* = *0.007; df* = *246.5; t* = *8.1; p* < *0.001; estimated fixed effects coefficient for Time* = *0.21; Std.Error* = *0.035; df* = *214.7; t* = *6.1; p* < *0.001; see* Supplementary Table S3), but not in the passive-breath condition (*Passive breath: estimated fixed effects coefficient for etCO2* = *–0.01; Std.Error* = *0.018; df* = *58.8; t* = *0.7; p* = *0.51; estimated fixed effects coefficient for Time* = *0.017; Std.Error* = *0.051; df* = *95.0; t* = *0.3; p* = *0.73; see* Supplementary Table S3). This relation between measurement time point, etCO_2_ and experience depth also becomes apparent when visualizing the correlation between etCO_2_ and experience depth progressively for each of our six measurement time points. As shown in Fig. 3A, etCO_2_ and experience depth are most tightly correlated around measurement time points 2–3, with a somewhat weaker relationship for time points 4–6. Interestingly, the relationship is weakest and fails to reach statistical significance at time point 1. Finally, etCO_2_ and experience depth appear to be more robustly related to each other in the active-breath than the passive-breath group over time (see Fig. 3A and Supplementary Fig. S3). Together, these findings are consistent with the idea that a decrease in etCO_2_ may be a trigger condition for ASC onset (see Discussion). Supplementary Fig. S3 shows the same relationships including all individual data points, as well as split by breathwork tradition. The virtual absence of individual data points in the upper right and lower left corners of these scatter plots suggests that (1) in the context of breathwork, intense ASCs rarely occur without at least some reduction in etCO_2_ (e.g. <50 mmHg) and (2) ordinary consciousness is difficult to maintain once etCO_2_ drops below a threshold of approximately 20 mmHg.

To further explore how the physiological and experiential effects of breathwork intersected over time, we jointly visualized the average dynamics of both parameters throughout the session. As shown in Fig. 3C, D, at the beginning of the session, etCO_2_ falls and experience depth rises largely in unison. However, in the second half of the session, etCO_2_ gradually returns to baseline, while experience depth persists for some time even once etCO_2_ begins to return to normal. Interestingly, the passive-breath group also showed qualitatively somewhat similar dynamics of etCO_2_ and experience depth as the active-breath group, but confined to a much smaller parameter space (Fig. 3C, D; Correlation between mean trajectories of depth ratings for passive and active breath groups; n = 6 observation time points for active and passive breath each; Holotropic: r = 0.81; *p* = 0.05; *conf.int(r)*_*low*_ = *–0.01; conf.int(r)*_*high*_ = *0.98;* Conscious-Connected: r = 0.74; *p* = 0.09; *conf.int(r)*_*low*_ = *–0.18; conf.int(r)*_*high*_ = *0.97*). For examples of how the same dynamics evolve in individual participants, see Supplementary Figs. S1, S3.

### Sustained psychological effects of breathwork

Next, we examined if breathwork gave rise to physiological and psychological changes beyond the session itself. Since the physiological and experiential dynamics triggered acutely within the session were so similar across the two breathwork formats studied here (see Figs. 1–3), we considered both formats as the same treatment for the purpose of studying follow-on effects. However, statistical comparisons between the sustained effects of Holotropic and Conscious-Connected breathwork are shown in Supplementary Table S6, and confirm that their follow-on effects were largely identical.

**Fig. 4:**
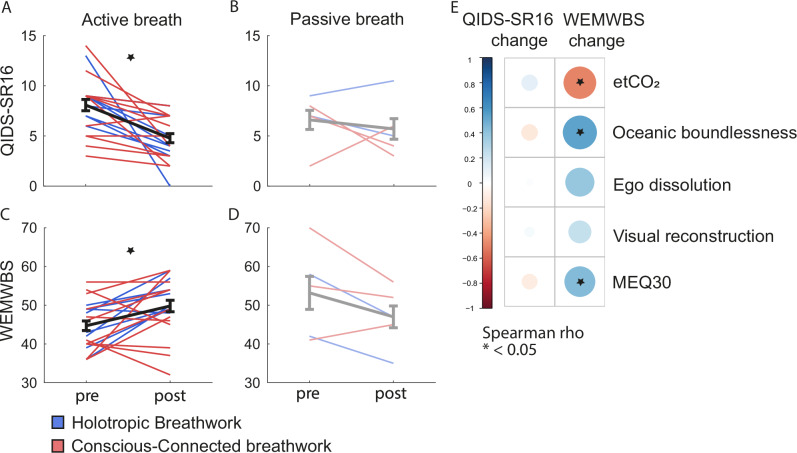
Psychological effects of breathwork. **A** Scores of the Self-Report Quick Inventory of Depressive Symptomatology (QIDS-SR16) one week before and after the breathwork session, completed by a total of 20 participants in the active-breath group. Red lines: Participants in Conscious-Connected breathwork (*n* = 12). Blue lines: Participants in Holotropic breathwork (*n* = 8). Black line and error bars: Mean ± SEM across participants. Star indicates statistically significant decrease in QIDS-SR16 scores at an Alpha level of 0.05. **B** Same as (**A**) for the passive-breath group that completed follow-on questionnaires. Light red: Conscious-Connected (*n* = 3 participants). Light blue: Holotropic (*n* = 2 participants). Grey line and error bars: Mean ± SEM. **C** Same as (**A**) for scores in the Warwick-Edinburgh Mental Wellbeing Scale (WEMWBS). Participant numbers as in (**A**). Star indicates statistically significant increase in WEMWBS scores at an Alpha level of 0.05. **D** Same as (**B**) for WEMWBS scores, including the same participant numbers. **E** Correlations between post-breathwork changes in QIDS-SR16 and WEMWBS scores on the one hand, and acute aspects of the breathwork session on the other hand, specifically average etCO_2_ during the active part of the session, as well as scores in the Mystical Experiences Questionnaire 30 (MEQ30) and the three subscales of the 11-Dimensional Altered States of Consciousness scale (11-DASC), which are termed ‘Oceanic boundlessness’, ‘Ego dissolution’ and ‘Visual reconstruction’. Correlation strength is colour-coded (see in-figure legend). Stars indicate statistically significant correlations. Correlations were computed based on all 25 participants who completed questionnaires one week before and after the breathwork session. Post-breathwork changes in QIDS-SR16 scores were not significantly predicted by any of the acute parameters of the breathwork session. In contrast, improvements in WEMWBS scores were predicted by lower etCO_2_ and by deeper ASCs during the session, indicated by both MEQ30 and 11-DASC scores.

To determine psychological follow-on effects of breathwork, in 33 participants we administered the self-report version of the 16-item Quick Inventory of Depressive Symptomatology (QIDS-SR16)^42^ and the Warwick-Edinburgh Mental Wellbeing Scale (WEMWBS)^43^ one week before and after the session. Of these, 25 participants completed the questionnaires at both time points. Both QIDS-SR16 and WEMWBS showed significant improvements post-session in the active-breath group (Fig. 4A, C; *paired t test; QIDS-SR16: df* = *19; t* = *4.4; Cohen´s D*_*4*_ = *1.29; bf01* = *0.01; p* < *0.001; WEMBWS: df* = *18; t* = *3.4; Cohen´s D*_*4*_ = *0.77; bf01* = *0.1; p* = *0.003; see* Supplementary Table S3). While the passive-breath group was unfortunately underpowered in terms of sample size, the observed data did not suggest a comparable improvement in QIDS-SR16 or WEMWBS scores (Fig. 4B,D; *paired t test; QIDS-SR16: df* = *4; t* = *0.6; Cohen´s D*_*4*_ = *0.27; bf01* = *2.6; p* = *0.61; WEMBWS: df* = *4; t* = *2.0; Cohen´s D*_*4*_ = *1.12; bf01* = *0.9; p* = *0.12; see* Supplementary Table S3).

To test if the follow-up improvements in mental well-being for the active-breath group were a direct result of the physiological and/or experiential processes they encountered throughout the session, we quantified how well changes in QIDS-SR16 and WEMBWS scores were predicted by (1) reduced etCO_2_ during the session, and (2) MEQ30 and 11-DASC scores. Figure 4E shows prominent links between acute session parameters and subsequent changes in well-being, as measured by the WEMWBS. Specifically, subacute increases in WEMWBS scores were predicted by lower etCO_2_ (*r* = *–0.49; conf.int(r)*_*low*_ = *–0.75; conf.int(r)*_*high*_ = *–0.13; df* = *23; p* = *0.011*) during the session, as well as by deeper ASCs, as reflected by higher scores in the MEQ30 (*r* = *0.44; ; conf.int(r)*_*low*_ = *0.03; conf.int(r)*_*high*_ = *0.71; df* = *23; p* = *0.031*) and in the ‘Oceanic boundlessness’ subscale of the 11-DASC (*r* = *0.54; ; conf.int(r)*_*low*_ = *0.17; conf.int(r)*_*high*_ = *0.76; df* = *23; p* = *0.006*).

### Subacute physiological effects of breathwork

To establish whether breathwork also caused subacute physiological shifts, we compared concentrations of two molecular markers in saliva directly before and after the breathwork session: The inflammatory marker interleukin-1 beta (IL-1β), and alpha-amylase (α-amylase), which is a marker of autonomic nervous system (ANS) activity, particularly its sympathetic branch^44,45^.

**Fig. 5:**
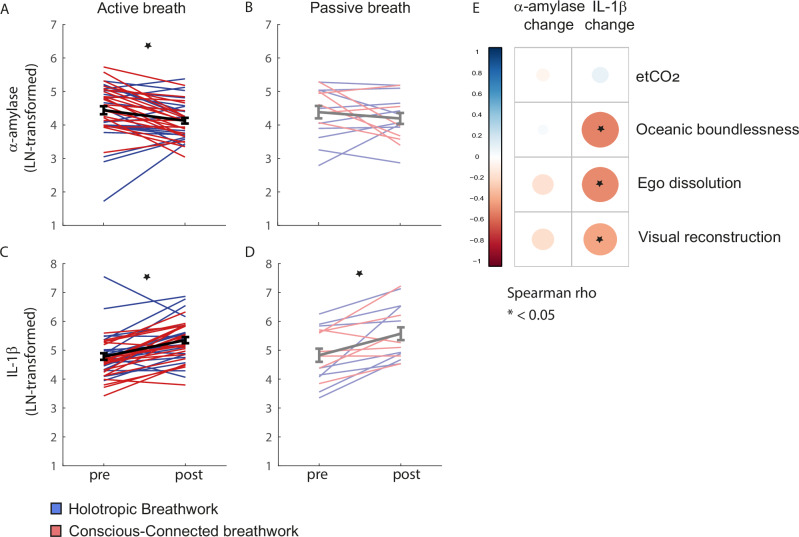
Subacute physiological effects of breathwork. **A** Concentration of the autonomic nervous system activity marker alpha-amylase (α-amylase) directly before and after the breathwork session, completed by a total of 40 participants in the active-breath group. Red lines: Participants in Conscious-Connected breathwork. Blue lines: Participants in Holotropic breathwork. Black line and error bars: Mean ± SEM across participants. Star indicates statistically significant decrease in α-amylase at an Alpha level of 0.05. **B** Same as (**A**) for passive-breath participants (*n* = 15). Light red: Conscious-Connected. Light blue: Holotropic. Grey line and error bars: Mean ± SEM. **C** Same as A for concentrations of the inflammatory marker interleukin-1 beta (IL-1β). Star indicates statistically significant increase in IL-1β at an Alpha level of 0.05. **D** Same as B for concentrations of IL-1β. Star indicates statistically significant increase in IL-1β at an Alpha level of 0.05. **E** Same as Fig. 4E for correlations between the acute aspects of the breathwork session and the post-breathwork changes in α-amylase and IL-1β levels. IL-1β increases were linked to subjective experiences, but not etCO_2_, during breathwork, such that deeper ASCs predicted smaller increases in IL-1β post-session. Change in α-amylase was not significantly predicted by any of the acute parameters of the breathwork session.

After the breathwork session, α -amylase levels decreased overall. Specifically, active breathers showed significantly lower levels of α-amylase post-session (*paired t test: df* = *39; t* = *3.4; Cohen´s D*_*4*_ = *0.88; bf01* = *0.1; p* = *0.001*; *see* Supplementary Table S3*for details and* see Supplementary Table S6 for comparison between breathwork formats), while the decrease was smaller and failed to reach significance in the passive-breath group (*paired t test: df* = *14; t* = *1.0; Cohen´s D*_*4*_ = *0.35; bf01* = *2.6; p* = *0.35; see* Supplementary Table S3) (Fig. 5A,B). In contrast, IL-1β levels increased post-session. In active breathers, this post-session increase in IL-1β was significant (Fig. 5C*; paired t test: df* = *39; t* = *5.2; Cohen´s D*_*4*_ = *0.88; bf01* < *0.001; p* < *0.001; see* Supplementary Table S3), and we also observed a smaller but statistically significant increase in passive breathers (Fig. 5D; *paired t test: df* = *14; t* = *4.1; Cohen´s D*_*4*_ = *0.92; bf01* = *0.03; p* = *0.001; see* Supplementary Table S3).

We wondered if these physiological follow-on effects were simply a result of the physiological processes set in motion during breathwork, or if they were also shaped by the subjective experiences participants encountered. To answer this question, we first related average etCO_2_ during the active phase of the session to subacute physiological changes. etCO_2_ did not predict physiological outcomes significantly (Fig. 5E). In contrast, subjective experience appeared to modulate physiological outcomes: All three subscales of the 11-DASC, ‘Oceanic boundlessness’ (*r* = *–0.49; df* = *54; p* < *0.001*), ‘Ego dissolution‘ (*r* = *–0.48; df* = *54; p* < *0.001*) and ‚Visual reconstruction‘ (*r* = *–0.48; df* = *54; p* = *0.003)*, showed negative correlations with IL-1β change, so that weaker ASCs predicted larger increases in inflammation post-session (Fig. 5E).

Together, these results indicate a scenario in which decreased etCO_2_ is a crucial factor in triggering strong ASCs during breathwork, and the subjective qualities of these ASCs in turn predict sustained outcomes, both in terms of the psychological and physiological effects of breathwork.

## Discussion

In this exploratory study, we have demonstrated that the subjective experiences triggered by circular breathwork bear a close resemblance to those evoked by psychedelics across experiential domains such as ego dissolution, unity and bliss. By tracking physiological and experiential dynamics throughout a breathwork session, we were able to show that while this effect appears to be partially supported by general context (including evocative music and social support), the physiological changes associated with circular breathwork, measured here by a reduction in CO_2_ pressure (etCO_2_), are instrumental in triggering such ASCs. The depth of the experienced ASCs in turn predicts subacute effects of breathwork on the psychological and physiological level. Finally, by comparing these effects in two popular and complementary formats of breathwork practice – Holotropic and Conscious-Connected breathwork—we could demonstrate that these different breathwork styles engage the same physiological mechanisms and produce comparable experiential and psychological outcomes.

### Subjective effects of breathwork

The subjective effects of breathwork in this study were measured with two classical self-report questionnaires used in psychedelic research: MEQ30 and 11D-ASC. Scores for both questionnaires were comparable to those expected in the context of psychedelic-augmented therapy, with 3 of 11 sub-scales of the 11D-ASC and all 4 sub-scales of the MEQ-30 scoring indistinguishable (according to Bayes Factors analysis) or higher compared to experiences typically evoked by standard therapeutic doses of psilocybin or LSD^54,55^ (see Fig. 2A, B and Database Analysis section of Methods). This outcome is particularly remarkable given the fact that participants’ experiences were repeatedly interrupted by our experimental measurements throughout the session—which presumably may have hampered experiences at least to some extent. These results are in line with the recently published work by^56^, but stronger than the effects reported by^10^. This suggests that similarly to psychedelic experiences, different breathwork settings may deliver different experiential qualities^57^. What’s more, self-reports one week post-session indicated improvements in general well-being and a reduction of depressive symptoms; and the depth of the acute breathwork experience predicted such positive subacute outcomes. This dynamic resembles results that have been reported for psychedelic-augmented therapy, where the intensity of mystical experiences is also associated with subsequent improvements in mental health outcomes^58^. This positions circular breathwork as a viable alternative in cases where psychedelic therapy may be indicated but not accessible.

How does breathwork evoke these stark subjective effects? Our study indicates that the physiological changes driven by deliberate hyperventilation, quantified here in terms of decreased etCO_2_, likely play a decisive role in facilitating ASCs during breathwork. Decreases in etCO_2_ were strongly linked to deeper ASCs (see Fig. 3A, B), and this relationship was strongest as participants began to enter the intense portion of the session (see Fig. 3C), further suggesting that falling etCO_2_ may act as a catalyst for participants to enter ASCs, which are then sustained with less direct reliance on physiological processes, explaining the weaker relation between etCO_2_ and ASC depth towards the second half of the session (see Fig. 3D, E). Finally, when considering all individual data points (see Supplementary Fig. S3), one can see that intense ASCs virtually never occurred unless etCO_2_ fell below standard levels of approx. 35 mmHg. Conversely, when etCO_2_ fell below approx. 20 mmHg, it was virtually guaranteed to trigger at least some (and often a strong) departure from ordinary waking consciousness. This effect is particularly intriguing because in non-breathwork-related circumstances, an etCO_2_ of 20 mmHg or less would be considered a sign of severe physiological malfunctions, e.g. of the heart or lungs^17,59^.

These observations tally with the concept of Pivotal Mental States introduced in 2021 by Brouwer and Carhart-Harris^60^. In this conceptual framework, unusual and overwhelming physiological challenges such as heat/cold exposure, food deprivation, sensory overload or pain can cause neuronal processing to transition into a state of heightened perception and learning. This switch is hypothesized to be mediated by a drastic upregulation of serotonin signalling^60^. As such, communal rituals like sweat lodges, traditional tattooing or religious fasting might be experiential ways to deliberately elicit pivotal mental states, while classical psychedelics would be a pharmacological avenue to ‘hijack’ the same innate system. By perturbing the normal equilibrium of blood gas concentrations, the physiological processes set in motion by breathwork fall squarely into the category of potential non-pharmacological triggers for such Pivotal Mental States. This notion is also consistent with our observation that reduced etCO_2_ (and its physiological follow-on effects) may serve as a ‘trigger condition’, initiating deep ASCs, which are then maintained for some time even when etCO_2_ has normalized again (see Fig. 3D, E). This dynamic could be explained by the fact that once neuronal processing has transitioned into a Pivotal Mental State, the initial physiological trigger for this switch is not required to remain present.

While a reduction in etCO_2_ appeared to be an important trigger condition for ASCs in this study, one should note that participants in the passive-breath condition also reported limited but significant deviations from every-day consciousness (see Fig. 2). This suggests that although physiological changes are closely linked to subjective experiences during breathwork, the broader session setting also plays an important role in shaping and enhancing subjective experiences^61^. To some extent, this effect is likely to be a true reflection of the role that context plays in a breathwork session. Such context factors include emotionally evocative music, social support by the group and facilitators both during the session itself and in the group sharing before the session, and potential ‘social contagion’ by the experiences of other session participants, who might e.g. express intense emotions by crying, screaming or laughing. In fact, such prominent context factors of breathwork are intentionally designed to evoke and support emotional processing (see^62^). As such, in our view the fact that the breathwork setting alone can alter consciousness to some extent does not put the effectiveness of the circular breathing technique itself in question. Rather, it reflects the fact that different breathwork schools have successfully developed contextual elements to enhance the processes that the breathing technique itself is meant to evoke. In addition, the setting effects measured in this study are likely heightened due to the fact that it was conducted in experienced breathwork practitioners. It stands to argue that simply due to their prior experience, participants were better able to shift their processing away from every-day consciousness when experiencing the (to them) familiar setting of a breathwork session. It is therefore likely that if the same experiential metrics were recorded in naive participants, the passive-breath group might report experience depths closer to ordinary consciousness than in this study – a hypothesis that should be tested in future experiments.

### Comparison between breathwork styles

Another interesting and perhaps unexpected aspect of our findings is just how much the effects of Holotropic and Conscious-Connected breathwork resemble each other, both in terms of within-session physiological and experiential dynamics (Figs. 1–3 and Supplementary Figs. S1 and S3), and of psychological and physiological follow-on effects (Figs. 4–5 and Supplementary Table S6). This similarity is particularly remarkable because we conducted both breathwork styles in the way they are most commonly practiced. This gives our measurements an ecological validity that we see as crucial, but it also means that, while both breathwork practices share the same core breathing technique, their overall format is not identical (see Table 1). This holds especially with regards to session duration: At a typical duration of three hours, Holotropic breathwork sessions last approximately twice as long as Conscious-Connected breathwork sessions. The fact that in-session dynamics are nevertheless so similar (see Fig. 3D, E) might be due to the participants‘ pre-existing expectations and experience of a specific timeline^62,63^, as well as the guidance from facilitators and the corresponding musical cues, which follow a similar overall dynamic (increasingly active breathing—emotional release—relaxation), but adjusted to the total session duration of each breathwork style. Beyond acute experiential dynamics, the fact that both breathwork styles had such similar short- and long-term effects despite differences in format and especially session duration also suggests that longer breathwork sessions may not equate to deeper experiences or more powerful long-term changes. This not only narrows the field of potential underlying mechanisms, but also has implications for the practical implementation of breathwork—given that in Holotropic breathwork, participants typically undergo two sessions – one as active participant and one as ‘sitter’ – equating to a time investment of at least six hours, compared to approx. two hours for a Conscious-Connected breathwork session.

### Sustained psychological effects of breathwork

Our self-report metrics also indicated improvements in overall well-being and depressive symptoms one week after the session (see Fig. 4). To some extent, these improvements might be ascribed to expectation effects, given that our participants were experienced breathwork practitioners, and thus likely to see breathwork as a beneficial activity (see section ‘Limitations’ below). On the other hand, one should also note that the measured subacute increases in well-being are in line with previous reports^8,10,11^, and improvements depended on ASC depth throughout the session—a relationship that has also been reported for psychedelic experiences^54^. Together, these results suggest that improvements in well-being were substantial beyond the contribution e.g. of placebo or expectation effects.

One interesting feature of our self-report metrics was that according to the scoring criteria of the QIDS-SR16, a portion of participants reported symptoms of mild, and in three cases moderate, depression one week before the breathwork session (see Fig. 4A, B). These somewhat elevated baseline levels of depressive symptoms might be explained in several ways. First, given the high prevalence of diagnosed depression^64^, it is reasonable to assume that mild levels of undiagnosed depression may be even more prevalent across the population. Beyond that, practices involving ASCs, including psychedelics and breathwork, have been reported to increase emotional openness^65–68^. As such, participants might simply be more aware of e.g. feelings of sadness or discomfort, and more willing to report them. Another intriguing possibility arises from the observation that one week post-session, most participants in the active-breath group reported decreased depressive symptoms (see Fig. 4A). As such, one could hypothesize that generally well-functioning persons with mild mental health disorders (such as mild depression) might take up breathwork as a form of self-medication, which might help them to alleviate symptoms for days, potentially weeks, post-session. Further experiments will be needed to disentangle these possibilities.

Finally, while most participants reported improvements in well-being, some also reported a decrease, suggesting potential adverse effects of breathwork in individual cases. In this study, participants had the opportunity to describe effects of their breathwork session in open questions both directly after the session and in the one-week follow-up. In these free-form questions, no specific adverse developments were mentioned. However, in future studies we would recommend to explore breathwork’s risk profile and potential adverse effects with more targeted questions.

By which mechanisms might breathwork promote sustained improvements in psychological well-being? As mentioned above, the conceptual framework of pivotal mental states might suggest that entering a highly unusual physiological state then engages a neuronal state change, altering and enhancing both perception and learning. However, even if this is correct, this mechanism does not determine the content of what is perceived and learned. What ‘lessons’ can we expect breathwork practitioners to encounter? First, considering the structure of typical breathwork interventions, we can speculate that they may provide the opportunity for participants to encounter corrective emotional experiences^69,70^, and to do so in a highly visceral way, for instance by feeling fully, and often physically, supported in vulnerable emotional processes - by facilitators, sitters and/or fellow participants. Bringing intense emotions to the surface, a consistent feature of breathwork experiences^7^, may also support participants in encountering potentially avoided emotions in a safe setting^12,15,71,72^, thereby reducing the need to engage in experiential avoidance^73–77^.

On a more abstract level, the co-existence of feelings of challenge and relaxation, of sympathetic and parasympathetic activation, which characterizes many breathwork experiences as well as psychedelic experiences^78^ has been interpreted as a hallmark of experiences of awe^79^. Feelings of awe can be evoked by a large variety of settings, e.g. psychedelic and mystical, spiritual and communal experiences, as well as encounters with nature or human community. Recent work has highlighted awe as a vital ingredient of human well-being^79^, and demonstrated its benefits e.g. for mental well-being, stress and conflict resolution, curiosity and community support^79–84^. Similar to psychedelic states, breathwork may constitute an intentional – and more readily available - access point to this beneficial emotional space.

### Subacute physiological effects of breathwork

In terms of physiological changes triggered by breathwork, the coexistence of decreased markers of sympathetic nervous system activity (α-amylase) but increased inflammatory immune responses (IL-1β) may seem contradictory at first glance, particularly since enhanced immune system activity would typically be thought to reflect increased rather than decreased stress. One potential explanation for this pattern is that by disturbing the normal equilibrium of blood gases, a breathwork session initially represents a temporary stressor to the body—and potential emotional releases during the session may act as a temporary psychological stressors^12,71^. This is likely to trigger activity across several stress response systems, particularly the sympathetic nervous system and hypothalamic-pituitary axis, which may in turn upregulate the immune system. Thus, we hypothesize that breathwork initially triggers the sympathetic nervous system and thereby activates the immune system, leading to an increase in IL-1β. As the session progresses, and participants return to relaxation, the ANS may then transition from sympathetic to parasympathetic activity, resulting in lower levels of α-amylase post-session. If true, this dynamic would be consistent with the notion of breathwork as a cathartic process, which involves experiencing and expressing challenging emotions and sensations in order to arrive at feeling of release and relaxation^12,15,85,86^

To test this theory, future studies should monitor biomarker profiles more comprehensively by tracking pro- and anti-inflammatory cytokines over a longer time frame, as well as throughout the session. It is also important to note that our analysis was based on saliva samples, where the status of mucosal immunity and overall mouth health may have contributed to the observed IL-1β levels. For future studies, it would be useful to measure plasma levels of cytokines as well as immune cell phenotypes to get a more comprehensive picture of how circular breathwork affects the immune system.

Interestingly, neither α-amylase nor IL-1β levels post-session were predicted by acute changes in etCO_2_ throughout the session (see Fig. 5E). Instead, IL-1β levels were modulated by participants’ subjective experiences: The further participants ventured away from every-day consciousness, the less their inflammatory markers increased post-session. This further supports the notion that the sustained effects of breathwork, both on the psychological and physiological level, are not the simple result e.g. of breathwork acting as a hormetic physiological stimulus, but directly depend on the subjective content of the experience itself (see^67,72,87–90^).

Finally, work on related breathing techniques, especially the Wim Hof Method, suggests that in the long run, engaging in circular breathwork may lead to a sustained decrease of sympathetic reactivity to mental stress^91^ and may ultimately act as an anti-inflammatory^21^. In line with previous results^21,23^, our findings suggest that while breathwork may have anti-inflammatory effects in the long term, during the session it appears to act as a temporary challenge to the system, increasing inflammation. Such seemingly paradoxical effects can be interpreted as processes of hormesis, where physical challenges such as fasting or physical workouts provide short-term stressors to the body that increase physical fitness long-term. Further longitudinal studies of breathwork and its psychological and physiological long-term effects will be needed to test these hypotheses.

### Limitations

While this study offers new insights into the experiential dynamics associated with breathwork, it also faces technical limitations that can hopefully be tackled in future studies. For instance, experiential depth ratings throughout the session were assigned such that the highest score was also recorded when participants did not respond at all. Scoring these two states separately would give a better differentiation of subjective experiences. In terms of post-session self-reports, we used classical questionnaires developed mainly to assess psychedelic experiences. While this is helpful to enable direct comparisons of breathwork to existing literature on ASCs, it may also miss out on experiential aspects that may be unique to breathwork compared to psychedelic experiences. As a complementary approach to such classical questionnaires, we therefore recommend to also collect free reports of breathwork experiences, and extract experiential dimensions in a data-driven way e.g. using deep learning approaches. Finally, so far experiential self-reports of any kind face the trade-off between disrupting the participant’s experience as it happens, or working with post-session reports, which may or may not reflect an accurate recollection of the experience—particularly in the context of ASCs, which are likely to e.g. distort participants’ sense of time. In future studies, a first simple step to account for this dilemma would be to ask participants to rate the degree of disruption that they experienced as a result of experimental measurements. Beyond self-reports, we would also propose to directly derive experiential states from behavioural analyses. If successful, this would remove disruptions for the participants while estimating experiential states at high temporal resolution—which in turn would allow for direct links to neuronal activity recorded simultaneously.

What’s more, although experiential and physiological dynamics during breathwork appear closely linked in this study, one should note that this link may be at least partially impacted by the fact that the study recruited experienced breathwork practitioners. Given their background, participants may have held the expectation (either based on their own previous breathwork experiences or on prevalent beliefs held by the broader breathwork community) that the more intense the hyperventilation, the more profound the resulting ASCs. If so, at least part of the observed relationship between etCO_2_ reduction and ASC onset could potentially be explained by a placebo effect^63,92^. Similarly, the improvements in overall well-being and depressive symptoms one week after the session reported here (see Fig. 4) may to some extent have been enhanced by prior convictions of the participants: Experienced breathwork practitioners are likely to perceive breathwork as a practice that generally enhances their quality of life. Therefore, they might be expecting to see improved well-being post-session (see^63,92^). To address these possibilities in future studies, it would be interesting to ask participants to report their beliefs about the relationship between breathing pattern and subjective breathwork experiences, as well as about the long-term effects of breathwork. Another option would be to conduct a complementary study in naïve participants, who would presumably not hold preconceived notions about the effects of breathwork. Such a study would however likely face different challenges, e.g. in terms of participants being more easily disturbed by measurements throughout the session.

Another interesting set of questions centres around the role of set and setting in breathwork experiences. Here, we explore the contribution of set and setting by exposing participants to the same session setting while varying their breathing technique across experimental conditions. In a follow-up study, the complementary manipulation – i.e. keeping the circular breathing technique constant, but varying typical context factors such as music and group sharing - could further reveal how central the circular breathing technique itself is in evoking ASCs in the absence of supportive context factors. On a more basic level, the sample size we were able to recruit here limits the conclusions we were able to draw regarding effects in the control (passive-breath) group. Especially for the follow-on questionnaires (QIDS and WEMBWS), and to some extent for the biomarker measurements we obtained, this group was too small to ascertain differences statistically (see Supplementary Table S2). Thus, future studies would require larger sample sizes to further determine the contribution of context effects to breathwork.

Finally, while the current study highlights potential benefits of breathwork, it is also important to note that potential adverse effects have not been sufficiently studied so far. In the current study we did not explicitly ask about adverse effects, but rather gave participants space for general feedback about the session. In this general feedback, no adverse effects were mentioned, but more targeted questions might have revealed negative or aversive effects that participants experienced. Moreover, since this study recruited experienced breathwork practitioners, it will by definition underestimate the likelihood of adverse events in the general population. As such, we recommend that in future, more clinically focused studies of breathwork explicitly survey potential negative effects of breathwork to determine their likely prevalence.

## Conclusions

In summary, our results indicate that breathwork can effectively enhance well-being. We found that such improvements appear to be supported both by physiological and experiential and psychosocial mechanisms, some of which resemble those engaged by psychedelic-augmented therapy. These parallels includes similar ASCs whose depth predicts subsequent improvements in well-being, as well as the modulating function of set and setting^57,62^. Consistently with the concept of pivotal mental states^60^, the unusual physiological dynamics induced by breathwork, including severely reduced etCO_2_, may be central in triggering deep subjective breathwork experiences, which in turn predict both physiological and psychological follow-on effects. We believe that these findings not only open up new insights into the functional mechanisms and potential applications of breathwork—they also identify physiological boundary conditions in which ASCs can arise in a non-pharmacological context.

## Supplementary information

Supplementary Information Havenith et al

Transparent Peer Review file

Reporting Summary

## Data Availability

All anonymised raw data included in this study (including Supplementary Information) are available in the following repository: https://github.com/zero-noise-lab/BreathworkCO2.

## Code availability

All custom-written analysis code are available in the following repository: https://github.com/zero-noise-lab/BreathworkCO2.

## References

[1] Goodwin, G. M. et al. Single-dose psilocybin for a treatment-resistant episode of major depression. *N. Engl. J. Med.* **387**, 1637–1648 (2022). 36322843 10.1056/NEJMoa2206443

[2] Raison, C. L. et al. Single-dose psilocybin treatment for major depressive disorder: A randomized clinical trial. *JAMA* **330**, 843–853 (2023). 37651119 10.1001/jama.2023.14530 PMC10472268

[3] Griffiths, R. R. et al. Psilocybin produces substantial and sustained decreases in depression and anxiety in patients with life-threatening cancer: A randomized double-blind trial. *J. Psychopharmacol. (Oxford, England)* **30**, 1181–1197 (2016). 10.1177/0269881116675513 PMC5367557 27909165

[4] Carhart-Harris, R. L. et al. Psilocybin with psychological support for treatment-resistant depression: six-month follow-up. *Psychopharmacol. (Berl.)* **235**, 399–408 (2018). 10.1007/s00213-017-4771-x PMC5813086 29119217

[5] Mitchell, J. M. et al. MDMA-assisted therapy for severe PTSD: A randomized, double-blind, placebo-controlled phase 3 study. *FOC* **21**, 315–328 (2023). 10.1176/appi.focus.23021011 PMC10316215 37404971

[6] Von Rotz, R. et al. Single-dose psilocybin-assisted therapy in major depressive disorder: A placebo-controlled, double-blind, randomised clinical trial. *eClinicalMedicine* **56**, 101809 (2023). 36636296 10.1016/j.eclinm.2022.101809 PMC9830149

[7] Fincham, G. W. et al. High ventilation breathwork practices: An overview of their effects, mechanisms, and considerations for clinical applications. *Neurosci. Biobehav. Rev.* **155**, 105453 (2023). 37923236 10.1016/j.neubiorev.2023.105453

[8] Bahi, C. et al. Effects of conscious connected breathing on cortical brain activity, mood and state of consciousness in healthy adults. *Curr. Psychol.*10.1007/s12144-023-05119-6 (2023).

[9] Banushi, B. et al. Breathwork interventions for adults with clinically diagnosed anxiety disorders: A scoping review. *Brain Sci.* **13**, 256 (2023). 36831799 10.3390/brainsci13020256 PMC9954474

[10] Uthaug, M. V., Mason, N. L., Havenith, M. N., Vancura, M. & Ramaekers, J. G. An experience with Holotropic Breathwork is associated with improvement in non-judgement and satisfaction with life while reducing symptoms of stress in a Czech-speaking population. *J. Psychedelic Stud.* **5**, 176–189 (2021).

[11] Fincham, G. W., Strauss, C., Montero-Marin, J. & Cavanagh, K. Effect of breathwork on stress and mental health: A meta-analysis of randomised-controlled trials. *Sci. Rep.* **13**, 432 (2023). 36624160 10.1038/s41598-022-27247-y PMC9828383

[12] Lalande, L., Bambling, M., King, R. & Lowe, R. Breathwork: An additional treatment option for depression and anxiety? *J. Contemp. Psychother.* **42**, 113–119 (2012).

[13] Balban, M. Y. et al. Brief structured respiration practices enhance mood and reduce physiological arousal. *CR Med.* **0**, 100895 (2023). 10.1016/j.xcrm.2022.100895 PMC9873947 36630953

[14] de Wit, P. A. J. M. & Moraes Cruz, R. Treating PTSD with connected breathing: A clinical case study and theoretical implications. *Eur. J. Trauma Dissoc.* **5**, 100152 (2021).

[15] Rhinewine, J. P. & Williams, O. J. Holotropic breathwork: The potential role of a prolonged, voluntary hyperventilation procedure as an adjunct to psychotherapy. *J. Altern. Comp. Med.* **13**, 771–776 (2007). 10.1089/acm.2006.6203 17931070

[16] Bednarczyk, E. M. et al. Hyperventilation-induced reduction in cerebral blood flow: Assessment by positron emission tomography. *DICP* **24**, 456–460 (1990). 2343589 10.1177/106002809002400501

[17] Inbar, O., Inbar, O., Zohar, H. & Ofir, D. Physiological responses during a single rebirthing (Breath work) session: Physiological responses during the rebirthing treatment. *Med Sci. Discov.* **9**, 347–354 (2022).

[18] Krapf, R., Caduff, P., Wagdi, P., Stäubli, M. & Hulter, H. N. Plasma potassium response to acute respiratory alkalosis. *Kidney Int.* **47**, 217–224 (1995). 7731149 10.1038/ki.1995.26

[19] Stäubli, M., Vogel, F., Bärtsch, P., Flückiger, G. & Ziegler, W. H. Hyperventilation-induced changes of blood cell counts depend on hypocapnia. *Eur. J. Appl Physiol. Occup. Physiol.* **69**, 402–407 (1994). 7875136 10.1007/BF00865403

[20] Tercero, J. et al. Effects on cerebral blood flow of position changes, hyperoxia, CO2 partial pressure variations and the Valsalva manoeuvre: A study in healthy volunteers. *Eur. J. Anaesthesiol.* **38**, 49–57 (2021). 33074942 10.1097/EJA.0000000000001356

[21] Kox, M. et al. Voluntary activation of the sympathetic nervous system and attenuation of the innate immune response in humans. *Proc. Natl. Acad. Sci. USA* **111**, 7379–7384 (2014). 24799686 10.1073/pnas.1322174111 PMC4034215

[22] Muzik, O., Reilly, K. T. & Diwadkar, V. A. “Brain over body”–A study on the willful regulation of autonomic function during cold exposure. *NeuroImage* **172**, 632–641 (2018). 29438845 10.1016/j.neuroimage.2018.01.067

[23] Zwaag, J., Naaktgeboren, R., van Herwaarden, A. E., Pickkers, P. & Kox, M. The effects of cold exposure training and a breathing exercise on the inflammatory response in humans: A pilot study. *Psychosom. Med.* **84**, 457 (2022). 35213875 10.1097/PSY.0000000000001065 PMC9071023

[24] Nuding, S. C. et al. Functional connectivity in raphé-pontomedullary circuits supports active suppression of breathing during hypocapnic apnea. *J. Neurophysiol.* **114**, 2162–2186 (2015). 26203111 10.1152/jn.00608.2015 PMC4600964

[25] Ainslie, P. N. & Duffin, J. Integration of cerebrovascular CO_2_ reactivity and chemoreflex control of breathing: mechanisms of regulation, measurement, and interpretation. *Am. J. Physiol. -Regulatory Integr. Comp. Physiol.* **296**, R1473–R1495 (2009). 10.1152/ajpregu.91008.2008 19211719

[26] Bullock, T., Giesbrecht, B., Beaudin, A. E., Goodyear, B. G. & Poulin, M. J. Effects of changes in end‐tidal PO _2_ and PCO _2_ on neural responses during rest and sustained attention. *Physiol. Rep.* **9**, e15106 (2021). 10.14814/phy2.15106 PMC8578925 34755481

[27] Ito, H., Kanno, I., Ibaraki, M., Hatazawa, J. & Miura, S. Changes in human cerebral blood flow and cerebral blood volume during hypercapnia and hypocapnia measured by positron emission tomography. *J. Cereb. Blood Flow. Metab.* **23**, 665–670 (2003). 12796714 10.1097/01.WCB.0000067721.64998.F5

[28] Kety, S. S. & Schmidt, C. F. The effects of active and passive hyperventilation on cerebral blood flow, cerebral oxygen consumption, cardiac output, and blood pressure of normal young men 1. *J. Clin. Invest.* **25**, 107–119 (1946). 21016304

[29] Kontos, H. A., Wei, E. P., Raper, A. J. & Patterson, J. L. Local mechanism of CO_2_ action of cat pial arterioles. *Stroke* **8**, 226–229 (1977). 15334 10.1161/01.str.8.2.226

[30] Mueller, S. M., Heistad, D. D. & Marcus, M. L. Total and regional cerebral blood flow during hypotension, hypertension, and hypocapnia. Effect of sympathetic denervation in dogs. *Circ. Res*. **41**, 350–356 (1977). 890889 10.1161/01.res.41.3.350

[31] Szabo, K. et al. Hypocapnia induced vasoconstriction significantly inhibits the neurovascular coupling in humans. *J. Neurol. Sci.* **309**, 58–62 (2011). 21831399 10.1016/j.jns.2011.07.026

[32] Posse, S. et al. In vivo measurement of regional brain metabolic response to hyperventilation using magnetic resonance: Proton echo planar spectroscopic imaging (PEPSI). *Magn. Reson. Med* **37**, 858–865 (1997). 9178236 10.1002/mrm.1910370609

[33] Terekhin, P. & Forster, C. Hypocapnia related changes in pain-induced brain activation as measured by functional MRI. *Neurosci. Lett.* **400**, 110–114 (2006). 16517071 10.1016/j.neulet.2006.02.040

[34] Wise, R. et al. Dynamic forcing of end-tidal carbon dioxide and oxygen applied to functional magnetic resonance imaging. *J. Cereb. Blood Flow. Metab. J. Int. Soc. Cereb. Blood Flow. Metab.* **27**, 1521–1532 (2007). 10.1038/sj.jcbfm.9600465 17406659

[35] Prugger, J., Derdiyok, E., Dinkelacker, J., Costines, C. & Schmidt, T. T. The Altered States Database: Psychometric data from a systematic literature review. *Sci. Data* **9**, 720 (2022). 36418335 10.1038/s41597-022-01822-4 PMC9684144

[36] Grof, S. *The Adventure of Self-Discovery: Dimensions of Consciousness and New Perspectives in Psychotherapy and Inner Exploration* (State Univ of New York Pr, New York, NY, 1988).

[37] Grof, S. & Grof, C. *Holotropic Breathwork, Second Edition: A New Approach to Self-Exploration and Therapy*. (SUNY Press, Albany, 2023).

[38] Havenith, M. & Nemri, A. *Atemkraft - Das Breathwork-Handbuch*. (Schattauer, 2025).

[39] Studerus, E., Gamma, A. & Vollenweider, F. X. Psychometric evaluation of the altered states of consciousness rating scale (OAV). *PLOS ONE* **5**, e12412 (2010). 20824211 10.1371/journal.pone.0012412 PMC2930851

[40] Barrett, F. S., Johnson, M. W. & Griffiths, R. R. Validation of the revised mystical experience questionnaire in experimental sessions with psilocybin. *J. Psychopharmacol.* **29**, 1182–1190 (2015). 26442957 10.1177/0269881115609019 PMC5203697

[41] MacLean, K. A., Leoutsakos, J.-M. S., Johnson, M. W. & Griffiths, R. R. Factor analysis of the mystical experience questionnaire: A study of experiences occasioned by the hallucinogen psilocybin. *J. Sci. Study Relig.* **51**, 721–737 (2012). 23316089 10.1111/j.1468-5906.2012.01685.x PMC3539773

[42] Rush, A. J. et al. An evaluation of the quick inventory of depressive symptomatology and the Hamilton rating scale for depression: A sequenced treatment alternatives to relieve depression trial report. *Biol. Psychiatry* **59**, 493–501 (2006). 16199008 10.1016/j.biopsych.2005.08.022 PMC2929841

[43] Tennant, R. et al. The Warwick-Edinburgh Mental Well-being Scale (WEMWBS): Development and UK validation. *Health Qual. Life Outcomes* **5**, 63 (2007). 18042300 10.1186/1477-7525-5-63 PMC2222612

[44] Ali, N. & Nater, U. M. Salivary alpha-amylase as a biomarker of stress in behavioral medicine. *Int. J. Behav. Med.* **27**, 337–342 (2020). 31900867 10.1007/s12529-019-09843-x PMC7250801

[45] Nater, U. M. & Rohleder, N. Salivary alpha-amylase as a non-invasive biomarker for the sympathetic nervous system: Current state of research. *Psychoneuroendocrinology* **34**, 486–496 (2009). 19249160 10.1016/j.psyneuen.2009.01.014

[46] Genser, B., Cooper, P. J., Yazdanbakhsh, M., Barreto, M. L. & Rodrigues, L. C. A guide to modern statistical analysis of immunological data. *BMC Immunol.* **8**, 27 (2007). 17963513 10.1186/1471-2172-8-27 PMC2234437

[47] Krekelberg, B. klabhub/bayesFactor: Bayes only. Zenodo 10.5281/zenodo.13744717 (2024).

[48] Cohen, J. *Statistical Power Analysis for the Behavioral Sciences*. (Routledge, 1988).

[49] Fritz, C. O., Morris, P. E. & Richler, J. J. Effect size estimates: Current use, calculations, and interpretation. *J. Exp. Psychol. Gen.* **141**, 2–18 (2012). 21823805 10.1037/a0024338

[50] Rosenthal, R., Cooper, H. & Hedges, L. & others. Parametric measures of effect size. *Handb. Res. Synth.* **621**, 231–244 (1994).

[51] Keselman, H. J., Algina, J., Lix, L. M., Wilcox, R. R. & Deering, K. N. A generally robust approach for testing hypotheses and setting confidence intervals for effect sizes. *Psychol. Methods* **13**, 110–129 (2008). 18557681 10.1037/1082-989X.13.2.110

[52] Howell, D. C. *Statistical Methods for Psychology*, *3rd Ed*. xviii, 693 (PWS-Kent Publishing Co, Boston, MA, US, 1992).

[53] Agadzhanyan, N. A., Panina, M. I., Kozupitsa, G. S. & Sergeev, O. S. Subjective and neurological manifestations of hyperventilation states of different intensities. *Hum. Physiol.* **29**, 448–452 (2003). 13677200

[54] Hirschfeld, T., Prugger, J., Majić, T. & Schmidt, T. T. Dose-response relationships of LSD-induced subjective experiences in humans. *Neuropsychopharmacol* **48**, 1602–1611 (2023). 10.1038/s41386-023-01588-2 PMC10516880 37161078

[55] Liechti, M. E., Dolder, P. C. & Schmid, Y. Alterations of consciousness and mystical-type experiences after acute LSD in humans. *Psychopharmacology* **234**, 1499–1510 (2017). 10.1007/s00213-016-4453-0 PMC5420386 27714429

[56] Bahi, C. et al. Effects of conscious connected breathing on cortical brain activity, mood and state of consciousness in healthy adults. *Curr. Psychol.* **43**, 10578–10589 (2024).

[57] Golden, T. L. et al. Effects of setting on psychedelic experiences, therapies, and outcomes: A rapid scoping review of the literature. *Curr. Top. Behav. Neurosci.* **56**, 35–70 (2022). 35138585 10.1007/7854_2021_298

[58] Griffiths, R. R., Richards, W. A., Johnson, M. W., McCann, U. D. & Jesse, R. Mystical-type experiences occasioned by psilocybin mediate the attribution of personal meaning and spiritual significance 14 months later. *J. Psychopharmacol.* **22**, 621–632 (2008). 18593735 10.1177/0269881108094300 PMC3050654

[59] Arena, R. & Sietsema, K. E. Cardiopulmonary exercise testing in the clinical evaluation of patients with heart and lung disease. *Circulation* **123**, 668–680 (2011). 21321183 10.1161/CIRCULATIONAHA.109.914788

[60] Brouwer, A. & Carhart-Harris, R. L. Pivotal mental states. *J. Psychopharmacol.* **35**, 319–352 (2021). 33174492 10.1177/0269881120959637 PMC8054165

[61] Rebecchini, L. Music, mental health, and immunity. *Brain Behav. Immun. Health* **18**, 100374 (2021). 34761245 10.1016/j.bbih.2021.100374 PMC8566759

[62] Hartogsohn, I. Set and setting, psychedelics and the placebo response: An extra-pharmacological perspective on psychopharmacology. *J. Psychopharmacol.* **30**, 1259–1267 (2016). 27852960 10.1177/0269881116677852

[63] Enck, P. & Zipfel, S. Placebo effects in psychotherapy: A framework. *Front. Psychiatry* **10**, 456 (2019). 10.3389/fpsyt.2019.00456 PMC6606790 31293462

[64] Otte, C. et al. Major depressive disorder. *Nat. Rev. Dis. Prim.* **2**, 1–20 (2016). 10.1038/nrdp.2016.65 27629598

[65] Erritzoe, D. et al. Recreational use of psychedelics is associated with elevated personality trait openness: Exploration of associations with brain serotonin markers. *J. Psychopharmacol.* **33**, 1068–1075 (2019). 30816797 10.1177/0269881119827891

[66] Lebedev, A. V. et al. LSD-induced entropic brain activity predicts subsequent personality change. *Hum. Brain Mapp.* **37**, 3203–3213 (2016). 27151536 10.1002/hbm.23234 PMC6867426

[67] MacLean, K. A., Johnson, M. W. & Griffiths, R. R. Mystical experiences occasioned by the hallucinogen psilocybin lead to increases in the personality domain of openness. *J. Psychopharmacol.* **25**, 1453–1461 (2011). 21956378 10.1177/0269881111420188 PMC3537171

[68] Wagner, M. T. et al. Therapeutic effect of increased openness: Investigating mechanism of action in MDMA-assisted psychotherapy. *J. Psychopharmacol.* **31**, 967–974 (2017). 28635375 10.1177/0269881117711712 PMC5544120

[69] Goldfried, M. R. The corrective experience: A core principle for therapeutic change. in *Transformation in psychotherapy: Corrective experiences across cognitive behavioral, humanistic, and psychodynamic approaches* 13–29 (American Psychological Association, Washington, DC, US, 2012). 10.1037/13747-002.

[70] Hill, C. E. et al. Corrective experiences in psychotherapy: Definitions, processes, consequences, and research directions. in *Transformation in psychotherapy: Corrective experiences across cognitive behavioral, humanistic, and psychodynamic approaches* 355–370 (American Psychological Association, Washington, DC, US, 2012). 10.1037/13747-017.

[71] Straton, D. Catharsis reconsidered. *Aust. N. Z. J. Psychiatry* **24**, 543–551 (1990). 2073231 10.3109/00048679009062911

[72] Walther, R. F. E. & van Schie, H. T. ‘Mind-Revealing’ psychedelic states: psychological processes in subjective experiences that drive positive change. *Psychoactives* **3**, 411–436 (2024).

[73] Henschel, A. V., Williams, J. L. & Hardt, M. M. The role of experiential avoidance and emotion regulation in DSM-5 posttraumatic stress symptomatology. *J. Loss Trauma* **26**, 527–539 (2021).

[74] Kumpula, M. J., Orcutt, H. K., Bardeen, J. R. & Varkovitzky, R. L. Peritraumatic dissociation and experiential avoidance as prospective predictors of posttraumatic stress symptoms. *J. Abnorm. Psychol.* **120**, 617–627 (2011). 21604826 10.1037/a0023927 PMC3170875

[75] Phaf, R. H., Mohr, S. E., Rotteveel, M. & Wicherts, J. M. Approach, avoidance, and affect: a meta-analysis of approach-avoidance tendencies in manual reaction time tasks. *Front. Psychol.* **5**, 378 (2014). 10.3389/fpsyg.2014.00378 PMC4021119 24847292

[76] Weaver, S. S. et al. Sacrificing reward to avoid threat: Characterizing PTSD in the context of a trauma-related approach-avoidance conflict task. *J. Abnorm. Psychol.* **129**, 457–468 (2020). 32437204 10.1037/abn0000528 PMC7393639

[77] Wolgast, M., Lundh, L.-G. & Viborg, G. Experiential avoidance as an emotion regulatory function: an empirical analysis of experiential avoidance in relation to behavioral avoidance, cognitive reappraisal, and response suppression. *Cogn. Behav. Ther.* **42**, 224–232 (2013). 23721612 10.1080/16506073.2013.773059

[78] Bonnelle, V. et al. Autonomic nervous system activity correlates with peak experiences induced by DMT and predicts increases in well-being. *J. Psychopharmacol.* **38**, 887–896 (2024). 10.1177/02698811241276788 PMC11512487 39301949

[79] Monroy, M. & Keltner, D. Awe as a pathway to mental and physical health. *Perspect. Psychol. Sci.* **18**, 309–320 (2023). 35994778 10.1177/17456916221094856 PMC10018061

[80] Gottlieb, S., Keltner, D. & Lombrozo, T. Awe as a scientific emotion. *Cogn. Sci.* **42**, 2081–2094 (2018). 10.1111/cogs.12648 30056628

[81] Keltner, D. & Haidt, J. Approaching awe, a moral, spiritual, and aesthetic emotion. *Cognit. Emot.* **17**, 297–314 (2003). 29715721 10.1080/02699930302297

[82] Lucht, A. & van Schie, H. T. The evolutionary function of awe: A review and integrated model of seven theoretical perspectives. *Emot. Rev.* **16**, 46–63 (2024).

[83] Piff, P. K., Dietze, P., Feinberg, M., Stancato, D. M. & Keltner, D. Awe, the small self, and prosocial behavior. *J. Personal. Soc. Psychol.* **108**, 883–899 (2015). 10.1037/pspi0000018 25984788

[84] Rudd, M., Vohs, K. D. & Aaker, J. Awe expands people’s perception of time, alters decision making, and enhances well-being. *Psychol. Sci.* **23**, 1130–1136 (2012). 22886132 10.1177/0956797612438731

[85] Russo, M. A., Santarelli, D. M. & O’Rourke, D. The physiological effects of slow breathing in the healthy human. *Breathe (Sheff.)* **13**, 298–309 (2017). 29209423 10.1183/20734735.009817 PMC5709795

[86] Weiss, B., Wingert, A., Erritzoe, D. & Campbell, W. K. Prevalence and therapeutic impact of adverse life event reexperiencing under ceremonial ayahuasca. *Sci. Rep.* **13**, 9438 (2023). 10.1038/s41598-023-36184-3 PMC10256717 37296197

[87] Griffiths, R. R., Richards, W. A., McCann, U. & Jesse, R. Psilocybin can occasion mystical-type experiences having substantial and sustained personal meaning and spiritual significance. *Psychopharmacology* **187**, 268–283 (2006). 16826400 10.1007/s00213-006-0457-5

[88] Kangaslampi, S. Association between mystical-type experiences under psychedelics and improvements in well-being or mental health – A comprehensive review of the evidence. *J. Psychedelic Stud.* **7**, 18–28 (2023).

[89] Ko, K., Knight, G., Rucker, J. J. & Cleare, A. J. Psychedelics, mystical experience, and therapeutic efficacy: A systematic review. *Front Psychiatry* **13**, 917199 (2022). 35923458 10.3389/fpsyt.2022.917199 PMC9340494

[90] Yaden, D. B., Goldy, S. P., Weiss, B. & Griffiths, R. R. Clinically relevant acute subjective effects of psychedelics beyond mystical experience. *Nat. Rev. Psychol.* **3**, 606–621 (2024).

[91] Fonkoue, I. T. et al. Eight weeks of device-guided slow breathing decreases sympathetic nervous reactivity to stress in posttraumatic stress disorder. *Am. J. Physiol. -Regulatory Integr. Comp. Physiol.* **319**, R466–R475 (2020). 10.1152/ajpregu.00079.2020 PMC7642907 32847397

[92] van Elk, M. & Fried, E. I. History repeating: guidelines to address common problems in psychedelic science. *Ther. Adv. Psychopharmacol.* **13**, 20451253231198466 (2023). 37766730 10.1177/20451253231198466 PMC10521293

