# Supplementary material for: Decreased CO2 saturation during circular breathwork supports emergence of altered states of consciousness
Source: Commun Psychol. 2025 Apr 13;3:59. doi: 10.1038/s44271-025-00247-0 (PMC11994804; doi:10.1038/s44271-025-00247-0)
Supplement: Supplementary file 1 — Supplementary Information Havenith et al [file 44271_2025_247_MOESM1_ESM.pdf]

Supplementary Information

Decreased CO_2 saturation during circular breathwork supports emergence of altered states of consciousness

Martha N. Havenith*[1 ++], Max Leidenberger*[2], Jelena Brasanac*[3,4], Mafalda Corvacho[2], Inês Carmo Figueiredo[2], Leonie Schwarz[2], Malin Uthaug[5,6,7], Simona Rakusa[2], Marijan Bernardic[2], Liliana Vasquez-Mock[2], Sergio Pérez Rosal[10], Robin Carhart-Harris[7,8,9], Stefan M. Gold[3,4,11,12], Henrik Jungaberle[2,13], Andrea Jungaberle[2,13]

(1) Zero-Noise Lab, Ernst Strüngmann Institute for Neuroscience, Frankfurt a.M., Germany; (2) MIND Foundation, Berlin, Germany; (3) Charité – Universitätsmedizin Berlin, Department of Psychiatry and Neuroscience, Campus Benjamin Franklin, Berlin, Germany; (4) Charité – Universitätsmedizin Berlin, Medical Department, Psychosomatic Medicine, Campus Benjamin Franklin, Berlin, Germany; (5) Department of Neuropsychology and Psychopharmacology, Faculty of Psychology and Neuroscience, Maastricht University, Maastricht, Netherlands; (6) Somnivore Pty. Ltd., Bacchus Marsh, Australia; (7) The Centre for Psychedelic Research, Department of Brain Sciences, Faculty of Medicine, Imperial College London, London, United Kingdom; (8) Weill Institute for Neurosciences, University of California San Francisco, San Francisco, USA; (9) Sandler Neurosciences Center, University of California San Francisco, San Francisco, USA; (10) Universitätsklinikum Ruppin-Brandenburg (11) Institute of Neuroimmunology and Multiple Sclerosis (INIMS), Universitätsklinikum Hamburg-Eppendorf, Hamburg, Germany; (12) German Center for Mental Health (DZPG), Campus Charité Mitte, Berlin, Germany, (13) OVID Clinics, Berlin, Germany

* These authors contributed equally

[++] Correspondence: Martha Havenith; martha.havenith@esi-frankfurt.de; Ernst-Strüngmann Institute for Neuroscience, Deutschordenstr. 46, 60528 Frankfurt, Germany

Contents

Supplementary Table S1: Distribution of self-reported gender, age and employment status across experimental groups

Supplementary Table S2: Number of participants included in different analyses

Supplementary Table S3. Statistical metrics for all reported results

Supplementary Table S4: Studies included in the database analysis of 11D-ASC scores.

Supplementary Table S5: Studies included in the database analysis of MEQ30 scores.

Supplementary Table S6. Comparisons between the sustained effects of Holotropic and Conscious-Connected breathwork

Supplementary Figure S1. Individual examples of end-tidal CO_2 pressure and experience depth ratings.

Supplementary Figure S2. Cross-validation of hand sign ratings with post-session ratings of altered states of consciousness.

Supplementary Figure S3. Mixed-model analysis of the relationship between experience depth and etCO2 over time for Holotropic and Conscious-Connected breathwork.

Supplementary Table S1: Distribution of self-reported gender, age and employment status across experimental groups.

	Holotropic (n=30)		*Conscious-Connected (n=31)*	
	active	*passive*	*active*	*passive*
Gender				
Female	12	7	11	4
Male	6	3	10	3
Non-binary				1
n/a	2		2	
Mean Age (SD)	33.17(5.70)	35.9(8.34)	29.71(6.11)	36.5(8.68)
n/a	2		2	
Employment				
Employed	9	5	9	4
Self-employed	5	4	6	2
In education	2	1	5	0
Unemployed	2	0	0	1
n/a	2		2	1

Supplementary Table S2: Number of participants included in different analyses

	Total	*Active*	*Passive*	*Holotropic*	*Conscious-Connected*
$etCO_2$	61	43	18	30	31
Subjective experience	61	43	18	30	31
QIDS/WEMBWS	25	20	5	11	14
Biomarkers	55	40	15	27	28

Supplementary Table S3. ***Statistical metrics for all reported results***

a) Mean $etCO_2$ across groups: Two-Way ANOVA active/passive breath x breathing style

**Outcome metric**	**df**	**F**	**p**	**Cohen's D**	**Bf01**	**Interpretation**
Active/passive CO2	Total: 60<br>Error: 57<br>Comparison: 1	111.4	<0.001	3.03<br>CI (low): 2.72<br>CI (high): 3.35	< 0.001	**Mean CO2 Active < Mean CO2 Passive**
Holotropic/ Con-connected CO2	Total: 60<br>Error: 57<br>Comparison: 1	0.0	0.91	0.14<br>CI (low): 0.01<br>CI (high): 0.28	3.4	**Mean CO2 Holotropic = Mean CO2 Con.-Con.**
Interaction	Total: 60<br>Error: 57<br>Comparison: 1	0.0	0.91	n.a.	n.a.	**No interaction**

b) Minimum $etCO_2$ across groups: Two-Way ANOVA active/passive breath x breathing style

**Outcome metric**	**df**	**F**	**p**	**Cohen's D**	**Bf01**	**Interpretation**
Active/passive CO2	Total: 60<br>Error: 57<br>Comparison: 1	119.0	< 0.001	3.12<br>CI (low): 2.79<br>CI (high): 3.44	< 0.001	**Min CO2 Active < Min CO2 Passive**
Holoropic/ Con-connected CO2	Total: 60<br>Error: 57<br>Comparison: 1	0.1	0.81	0.06<br>CI (low): -0.07<br>CI (high): 0.20	3.7	**Min CO2 Holotropic = Min CO2 Con.-Con.**
Interaction	Total: 60<br>Error: 57<br>Comparison: 1	0.2	0.64	n.a.	n.a.	No interaction

c) Mean rating of experience depth across groups: Two-Way ANOVA active/passive breath x breathing style

Outcome metric	df	F	p	Cohen's D	Bf01	Interpretation
Active/passive depth rating	Total: 60<br>Error: 57<br>Comparison: 1	20.8	< 0.001	1.31<br>CI (low): 1.12<br>CI (high): 1.50	0.001	**Mean depth (active) > Mean depth (passive)**
Holotropic/ Con-connected depth rating	Total: 60<br>Error: 57<br>Comparison: 1	4.8	0.03	0.56<br>CI (low): 0.42<br>CI (high): 0.70	0.5	Mean depth (Holotr) ≈< Mean depth (Con.Con)
Interaction	Total: 60<br>Error: 57<br>Comparison: 1	0.4	0.53	n.a.	n.a.	n.a.

d) Maximum rating of experience depth across groups: Two-Way ANOVA active/passive breath x breathing style

Outcome metric	df	F	p	Cohen's D	Bf01	Interpretation
Active/passive depth rating	Total: 60<br>Error: 57<br>Comparison: 1	15.0	< 0.001	1.12<br>CI (low): 0.95<br>CI (high): 1.31	0.01	**Max depth (active) > Max depth (passive)**
Holotropic/ Con-connected depth rating	Total: 60<br>Error: 57<br>Comparison: 1	5.1	0.03	0.54<br>CI (low): 0.41<br>CI (high): 0.68	0.59	Max depth (Holotr) ≈< Max depth (Con.Con)
Interaction	Total: 60<br>Error: 57<br>Comparison: 1	0.95	0.33	n.a.	n.a.	n.a.

e) 11-DASC scores active-breathwork group scores minus **placebo** reference scores: t-tests for difference from zero, across 11 sub-scales

**Outcome metric**	**df**	**t**	**p**	**Cohen's D**	**Bf01**	**Interpretation**
Unity	42	13.9	< 0.001	n.a. (since there is only one group's standard deviation)	< 0.001	**Breathwork > Placebo**
Spiritual	42	6.4	< 0.001	-	< 0.001	**Breathwork > Placebo**
Bliss	42	13.1	< 0.001	-	< 0.001	**Breathwork > Placebo**
Insight	42	10.6	< 0.001	-	< 0.001	**Breathwork > Placebo**
Disembodiment	42	10.3	< 0.001	-	< 0.001	**Breathwork > Placebo**
Impaired Control	42	9.9	< 0.001	-	< 0.001	**Breathwork > Placebo**
Anxiety	42	12.1	< 0.001	-	< 0.001	**Breathwork > Placebo**
Complex Imagery	42	9.3	< 0.001	-	< 0.001	**Breathwork > Placebo**
Simple Imagery	42	9.0	< 0.001	-	< 0.001	**Breathwork > Placebo**
Synaesthesia	42	11.2	< 0.001	-	< 0.001	**Breathwork > Placebo**
Changed meaning	42	13.2	< 0.001	-	< 0.001	**Breathwork > Placebo**

f) MEQ scores active-breathwork versus placebo reference scores: t-tests across 11 sub-scales

Outcome metric	df	t	p	Cohen's D	Bf01	Interpretation
Transcendence	21	12.9	< 0.001	n.a. (see above)	< 0.001	**Breathwork > Placebo**
Positive Mood	21	12.1	< 0.001	-	< 0.001	**Breathwork > Placebo**
Ineffability	21	14.0	< 0.001	-	< 0.001	**Breathwork > Placebo**
Mystical Experience	21	10.2	< 0.001	-	< 0.001	**Breathwork > Placebo**

g) 11-DASC scores active-breathwork minus **psilocybin** reference scores: t-tests for difference from zero, across 11 sub-scales

Outcome metric	df	t	p	Cohen's D	Bf01	Interpretation
Unity	42	4.1	< 0.001	n.a. (see above)	0.01	Breathwork < Psilocybin
Spiritual	42	10.8	< 0.001	-	< 0.001	Breathwork < Psilocybin
Bliss	42	10.2	< 0.001	-	< 0.001	Breathwork < Psilocybin
Insight	42	1.2	0.25	-	**3.26**	**Breathwork = Psilocybin**
Disembodiment	42	3.5	< 0.001	-	0.03	Breathwork < Psilocybin
Impaired Control	42	2.2	0.03	-	0.67	Breathwork < Psilocybin
Anxiety	42	**-5.6**	< 0.001	-	**< 0.001**	**Breathwork > Psilocybin**
Complex Imagery	42	7.9	< 0.001	-	< 0.001	Breathwork < Psilocybin
Simple Imagery	42	10.6	< 0.001	-	< 0.001	Breathwork < Psilocybin
Synaesthesia	42	4.5	< 0.001	-	0.002	Breathwork < Psilocybin
Changed meaning	42	1.6	0.12	-	**1.89**	**Breathwork = Psilocybin**

h) MEQ scores active-breathwork versus **psilocybin** reference scores: t-tests across 11 sub-scales

Outcome metric	df	t	p	Cohen's D	Bf01	Interpretation
Transcendence	21	-0.1	0.91	n.a. (see above)	4.7	**Breathwork = Psilocybin**
Positive Mood	21	-0.1	0.94	-	4.7	**Breathwork = Psilocybin**
Ineffability	21	0.3	0.74	-	4.5	**Breathwork = Psilocybin**
Mystical Experience	21	0.7	0.50	-	3.8	**Breathwork = Psilocybin**

i) 11-DASC scores active-breathwork versus **LSD** reference scores: t-tests across 11 sub-scales

Outcome metric	df	t	p	Cohen's D	Bf01	Interpretation
Unity	42	4.1	< 0.001	n.a. (see above)	0.01	Breathwork < LSD
Spiritual	42	4.5	< 0.001	-	0.003	Breathwork < LSD
Bliss	42	5.1	< 0.001	-	< 0.001	Breathwork < LSD
Insight	42	**0.9**	0.38	-	**4.16**	**Breathwork = LSD**
Disembodiment	42	7.0	< 0.001	-	< 0.001	Breathwork < LSD
Impaired Control	42	4.4	< 0.001	-	0.003	Breathwork < LSD
Anxiety	42	**-6.6**	< 0.001	-	< 0.001	**Breathwork > LSD**
Complex Imagery	42	12.0	< 0.001	-	< 0.001	Breathwork < LSD
Simple Imagery	42	13.9	< 0.001	-	< 0.001	Breathwork < LSD
Synaesthesia	42	12.8	< 0.001	-	< 0.001	Breathwork < LSD
Changed meaning	42	**1.1**	0.27	-	**3.39**	**Breathwork = LSD**

j) MEQ scores active-breathwork versus **LSD** reference scores: t-tests across 11 sub-scales

**Outcome metric**	**df**	**t**	**p**	**Cohen's D**	**Bf01**	**Interpretaton**
Transcendence	21	0.04	0.97	n.a. (see above)	**4.7**	**Breathwork = LSD**
Positive Mood	21	**-5.0**	< 0.001	-	0.002	**Breathwork > LSD**
Ineffability	21	0.6	0.54	-	**3.9**	**Breathwork = LSD**
Mystical Experience	21	**-3.8**	0.001	-	0.03	**Breathwork > LSD**

k) 11-DASC scores active-breathwork versus **MDMA** reference scores: t-tests across 11 sub-scales

**Outcome metric**	**df**	**t**	**p**	**Cohen's D**	**Bf01**	**Interpretation**
Unity	42	4.6	< 0.001	n.a. (see above)	0.002	**Breathwork > MDMA**
Spiritual	42	4.2	< 0.001	-	0.01	**Breathwork > MDMA**
Bliss	42	-4.1	< 0.001	-	0.01	**Breathwork > MDMA**
Insight	42	6.5	< 0.001	-	< 0.001	**Breathwork > MDMA**
Disembodiment	42	5.2	< 0.001	-	< 0.001	**Breathwork > MDMA**
Impaired Control	42	4.1	< 0.001	-	0.01	**Breathwork > MDMA**
Anxiety	42	10.6	< 0.001	-	< 0.001	**Breathwork > MDMA**
Complex Imagery	42	5.9	< 0.001	-	< 0.001	**Breathwork > MDMA**
Simple Imagery	42	6.7	< 0.001	-	< 0.001	**Breathwork > MDMA**
Synaesthesia	42	9.8	< 0.001	-	< 0.001	**Breathwork > MDMA**
Changed meaning	42	6.6	< 0.001	-	< 0.001	**Breathwork > MDMA**

l) MEQ scores active-breathwork versus MDMA reference scores: t-tests across 11 sub-scales

Outcome metric	df	t	p	Cohen's D	Bf01	Interpretation
Transcendence	21	11.5	< 0.001	n.a. (see above)	< 0.001	**Breathwork > MDMA**
Positive Mood	21	9.2	< 0.001	-	< 0.001	**Breathwork > MDMA**
Ineffability	21	10.2	< 0.001	-	< 0.001	**Breathwork > MDMA**
Mystical Experience	21	9.4	< 0.001	-	< 0.001	**Breathwork > MDMA**

m) 11-DASC scores across groups: Two-Way ANOVA active/passive breath x 11-DASC sub-scale

Outcome metric	df	F	p	Cohen's D	Bf01	Interpretation
Active/passive 11DASC score	1 (total: 670)	94.0	< 0.001	0.77 CI (low): 0.75 CI (high): 0.79	0.1	**11DASC (active) > 11DASC (passive)**
11 sub-scales 11DASC score	10 (total: 670)	6.4	< 0.001	0.42 ± 0.35 CI (low): 0.35±0.35 CI (high): 0.49±0.36	2.0 ± 2.0	11DASC subscales may differ from each other
Interaction	10 (total: 670)	1.4	0.19	n.a.	n.a.	n.a.

n) MEQ scores across groups: Two-Way ANOVA active/passive breath x MEQ sub-scale

**Outcome metric**	**df**	**F**	**p**	**Cohen's D**	**Bf01**	**Interpretation**
Active/passive MEQ score	1 (total: 119)	19.0	< 0.001	0.22<br>CI (low): 0.16<br>CI (high): 0.29	0.4	**MEQ (active) > MEQ (passive)**
4 sub-scales MEQ score	3 (total: 119)	2.4	0.08	0.37±0.25<br>CI (low): 0.25±0.25<br>CI (high): 0.50±0.25	1.8 ± 1.6	MEQ subscales do not differ from each other
Interaction	3 (total: 119)	0.7	0.53	n.a.	n.a.	n.a.

o) 11-DASC scores passive-breathwork versus placebo reference scores: t-tests across 11 sub-scales

**Outcome metric**	**df**	**t**	**p**	**Cohen's D**	**Bf01**	**Interpretation**
Unity	42	7.3	< 0.001	n.a. (see above)	< 0.001	**Passive Breathwork > Placebo**
Spiritual	42	2.5	0.02	-	0.4	**Passive Breathwork > Placebo**
Bliss	42	5.0	< 0.001	-	0.004	**Passive Breathwork > Placebo**
Insight	42	3.5	0.002	-	0.1	**Passive Breathwork > Placebo**
Disembodiment	42	5.4	< 0.001	-	0.002	**Passive Breathwork > Placebo**
Impaired Control	42	5.1	< 0.001	-	0.003	**Passive Breathwork > Placebo**
Anxiety	42	4.8	< 0.001	-	0.01	**Passive Breathwork > Placebo**

Complex Imagery	42	3.3	0.004	-	0.1	**Passive Breathwork > Placebo**
Simple Imagery	42	4.3	< 0.001	-	0.02	**Passive Breathwork > Placebo**
Synaesthesia	42	3.3	0.004	-	0.1	**Passive Breathwork > Placebo**
Changed meaning	42	4.2	< 0.001	-	0.02	**Passive Breathwork > Placebo**

p) MEQ scores passive-breathwork versus placebo reference scores: t-tests across 11 sub-scales

**Outcome metric**	**df**	**t**	**p**	**Cohen's D**	**Bf01**	**Interpretation**
Transcendence	7	5.4	0.001	n.a. (see above)	0.02	**Passive Breathwork > Placebo**
Positive Mood	7	5.4	0.001	-	0.02	**Passive Breathwork > Placebo**
Ineffability	7	4.1	0.005	-	0.1	**Passive Breathwork > Placebo**
Mystical Experience	7	5.6	< 0.001	-	0.02	**Passive Breathwork > Placebo**

q) $etCO_2$ and depth rating linear mixed model

Outcome metric	Coefficient Estimate (b)	95% CI	Std.Error	df	t-value	p-value	Interpretation
Active breath $etCO_2$	-0.0618	[-0.0769, -0.0466]	0.007	246.5	-8.05	< 0.001	Significant negative effect
Active breath Time	0.2163	[0.1466, 0.2853]	0.035	214.7	6.12	< 0.001	Significant positive effect
Passive breath $etCO_2$	-0.01	[-0.0482, 0.0237]	0.018	58.8	0.7	0.51	Non-significant (CI includes zero)
Passive breath Time	0.017	[-0.0827, 0.1183]	0.051	95	0.3	0.73	Non-significant (CI includes zero)

r) QIDS scores pre-post. Paired t-test

Outcome metric	df	t	p	Cohen's D_4	Bf01	Interpretation
QIDS pre-post (active-breath)	19	4.3	< 0.001	1.03	0.01	**QIDS (active, post) < QIDS (active, pre)**
QIDS pre-post (passive-breath)	4	0.6	0.61	0.27	2.6	QIDS (passive, post) = QIDS (passive, pre)

s) WEMBWS scores pre-post. Paired t-test

Outcome metric	df	t	p	Cohen's D_4	Bf01	Interpretation
WEMBWS pre-post (active-breath)	18	3.4	0.003	0.77	0.1	**WEMBWS (active, post) > WEMBWS (active, pre)**
WEMBWS pre-post (passive-breath)	4	2.0	0.12	1.12	0.9	WEMBWS (passive, post) ≈ WEMBWS (passive, pre)

t) Alpha-amylase scores pre-post in active-breath group. Paired t-test

Outcome metric	df	t	p	Cohen's D_4	Bf01	Interpretation
Amylase pre-post (active-breath)	39	3.3	0.002	0.88	0.1	**Amylase (active, post) < Amylase (active, pre)**
Amylase pre-post (passive-breath)	14	1.0	0.34	0.35	2.6	Amylase (passive, post) = Amylase (passive, pre)

u) IL-1b scores pre-post in active-breath group. Paired t-test

Outcome metric	df	t	p	Cohen's D_4	Bf01	Interpretation
IL-1b pre-post (active-breath)	39	5.8	< 0.001	1.50	< 0.001	**IL1b (active, post) > IL1b (active, pre)**
IL-1b pre-post (passive-breath)	14	4.1	0.001	0.92	0.03	IL1b (passive, post) > IL1b (passive, pre)

Supplementary Table S4: Studies included in the database analysis of 11D-ASC scores.
Studies found in Altered States Database (ASDB; https://alteredstatesdb.org/; see Prugger et al., 2022)

	Study	Participants	Treatment
**Placebo**	(Carhart-Harris et al., 2016)	20	Saline
	(Schmidt et al., 2012)	19	Saline + Glucose
	(Murray et al., 2022)	18	Water
**Psilocybin**	(Carhart-Harris et al., 2018)	20	25 mg
	(Smigielski et al., 2019)	20	0.315mg/kg
	(Madsen et al., 2019)	2	24mg
	(Carbonaro et al., 2018)	20	20mg/70kg
	(Smigielski et al., 2020)	17	0.230mg/kg
**LSD**	(Carhart-Harris et al., 2016)	20	0.075 mg intravenous
	(Liechti et al., 2017)	24	0.1 mg oral
	(Preller et al., 2017)	22	0.1 mg oral
	(Holze et al., 2020)	28	0.1 mg oral
	(Holze et al., 2021)	16	0.1 mg oral
	(Schmid et al., 2021)	24	0.1 mg oral
**MDMA**	(Hysek at al., 2012a)	16	0.125 mg oral
	(Hysek et al., 2012b)	16	0.125 mg oral
	(Hysek et al., 2013)	16	0.125 mg oral
	(Schmid et al., 2021)	9	0.125 mg oral
	(Schmid et al., 2021)	18	0.125 mg oral

Supplementary Table S5: Studies included in the database analysis of MEQ30 scores.

	Study	Participants	Treatment
**Placebo**	(Wießner et al., 2023)	24	Alcohol solution
	(Carbonaro et al., 2018)	20	Lactose
**Psilocybin**	(Barsuglia et al., 2018)	18	20 mg / 70kg
	(Griffiths et al., 2016)	50	0.22-0.3mg/kg
	(Nicholas et al., 2018)	12	0.3mg/kg
	(Carbonaro et al., 2018)	20	20mg/70kg
**LSD**	(Holze et al., 2020)	28	0.1 mg oral
	(Holze et al., 2021)	16	0.1 mg oral
	(Schmid et al., 2021)	24	0.1 mg oral
	(Schmid et al., 2021)	11	0.1 mg oral
**MDMA**	(Holze et al., 2020)	28	0.125 mg oral
	(Schmid et al., 2021)	9	0.125 mg oral
	(Schmid et al., 2021)	18	0.125 mg oral

Supplementary Table S6. Comparisons between the sustained effects of Holotropic and Conscious-Connected breathwork.

All comparisons between the sustained effects of Holotropic and Conscious-Connected breathwork were tested with t-tests (ttest2 in MATLAB), taking into account only differences between the two active-breath groups (since this is where sustained effects were initially detected). Note that the only significant difference was found in the change of amylase levels, where Holotropic breathwork triggered a smaller decrease in amylase levels post-session. For all other comparisons, no significant differences were found, and the corresponding bf01 values indicated a robust likelihood that outcomes of holotropic and conscious-connected breathwork sessions came from the same distribution.

**Comparison**	**Means and t-test results**
WEMBWs pre-post change	df = 18; t = 1.28; p = 0.21<br>Mean ± SD: HB: 7.9±5.5 ; CCB: 3.3±9.1;<br>Cohen's D: 0.60 CI (low): 0.25 CI (high): 0.85<br>Bf01 = 1.49
QIDS-SR16 pre-post change	df = 18; t = 0.95; p = 0.35<br>Mean ± SD: HB: -4.2±3.8 ; CCB: -2.7±3.1;<br>Cohen's D: 0.4 CI (low): 0.05 CI (high): 0.76<br>Bf01 = 1.95
α-amylase pre-post change	df = 39; t = 3.20; p < 0.003<br>Mean ± SD: HB: -10.5±43.5 ; CCB: -58.0±50.4;<br>Cohen's D: 1.0 CI (low): 0.61 CI (high): 1.39<br>Bf01 = 0.08
IL-1β pre-post change	df = 39; t = 0.49; p = 0.63<br>Mean ± SD: HB: 146.5±196.5 ; CCB: 122.9±104.9;<br>Cohen's D: 0.2 CI (low): -0.15 CI (high): 0.54<br>Bf01 = 3.22

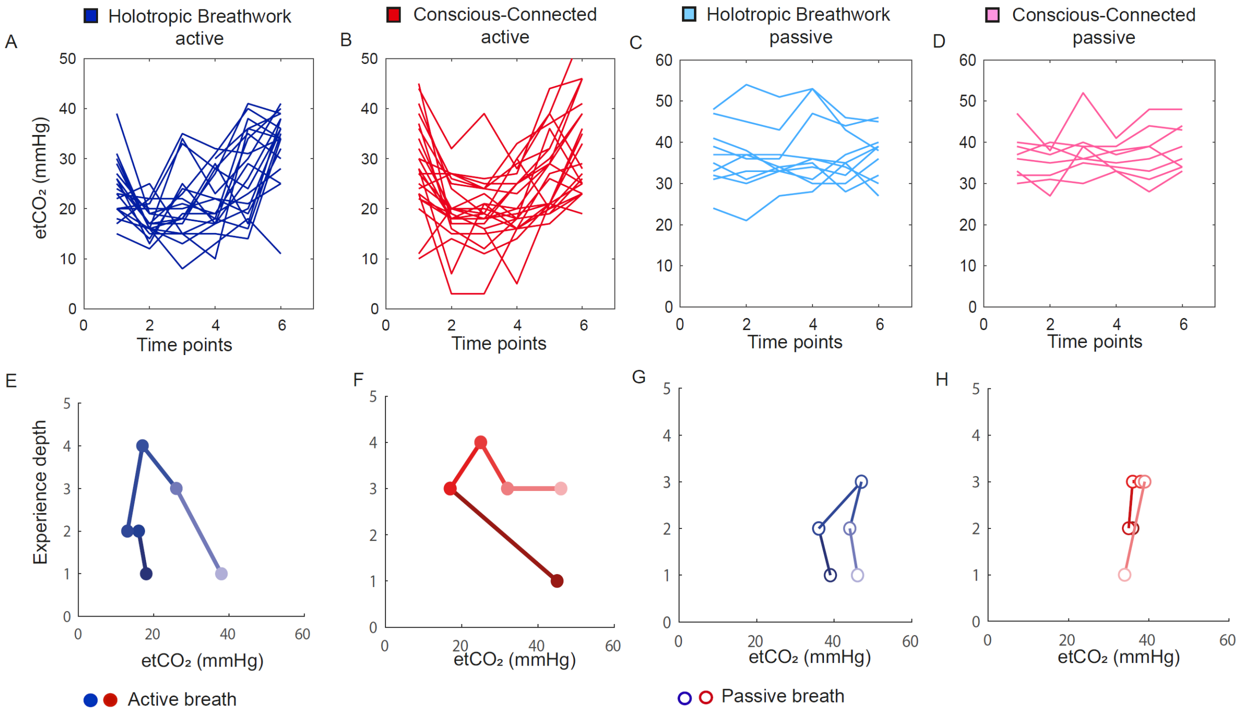

Supplementary Figure S1. Individual examples of end-tidal CO_2 pressure and experience depth ratings. (A) Individual time courses of end-tidal CO_2 pressure ($etCO_2$) across the six measurement time points of a Holotropic breathwork session. Each line represents one participant in the active-breath group. (B) Same as A for Conscious-Connected breathwork. (C) Same as A but for passive breathers in the Holotropic breathwork sessions. (D) Same as C for Conscious-Connected breathwork. (E) Trajectory of one example participant from the active-breath group in the Holotropic breathwork session (participant ID-11). Trajectories are depicted in the same way as in Figures 3C and D, relating $etCO_2$ (x axis) and experience depth (y axis) to each other over the six measurement time points of a session. (F) Same as E for the Conscious-Connected breathwork sessions (participant ID-38). (G) Same as E for a participant from the passive-breath group (participant ID-21). (H) Same as G for the Conscious-Connected breathwork sessions (participant ID-48).

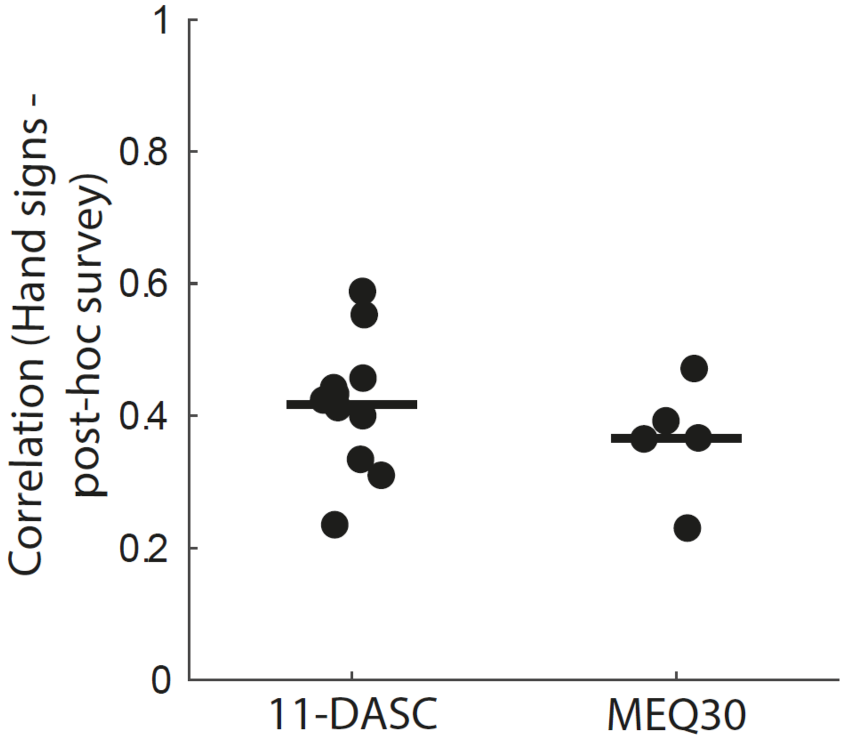

Supplementary Figure S2. Cross-validation of hand sign ratings with post-session ratings of altered states of consciousness. Correlation between average experience depth, as indicated by hand signs during the active part of the session (time points 2-4), and retrospective measures of altered consciousness by 11-Dimensional Altered States of Consciousness (11-DASC) and Mystical Experiences Questionnaire 30 (MEQ30) sub-scales. Left: Correlation coefficients with the 11 sub-scales of the 11DASC. Right: Correlation coefficients with the 4 sub-scales as well as overall score of the MEQ30. Horizontal lines: Average correlation coefficient across all sub-scales per questionnaire. 11-DASC: Mean r ± SEM = 0.42 ± 0.01; $p < 0.05$ in 10 of 11 sub-scales based on the family-wise error rate given by the Dunn-Sidak correction for multiple comparisons: MEQ: Mean r ± SEM = 0.37 ± 0.03; $p < 0.05$ in 3 of 4 sub-scales based on the family-wise error rate given by the Dunn-Sidak correction.

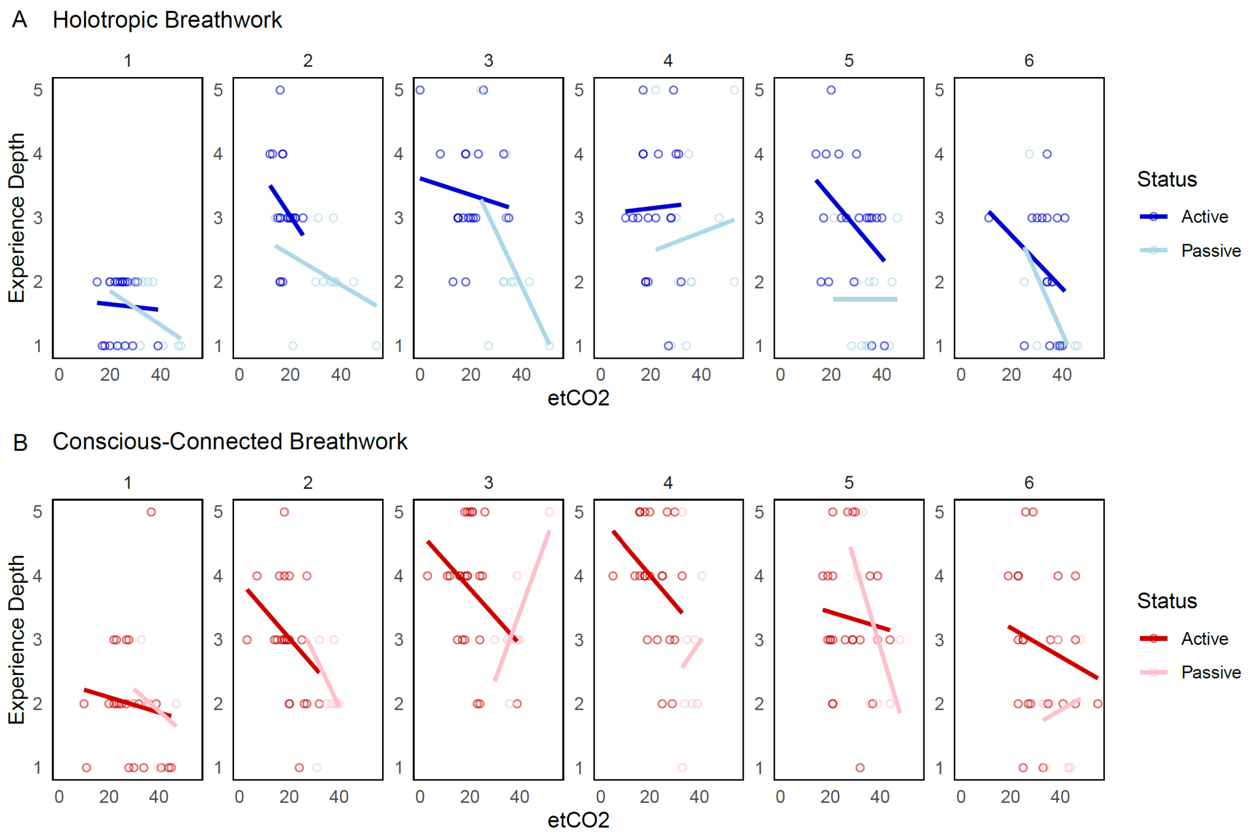

Supplementary Figure S3. Mixed-model analysis of the relationship between experience depth and etCO2 over time for Holotropic and Conscious-Connected breathwork.

In both breathwork techniques results indicated etCO2 and Time as significant predictors of Experience depth. Holotropic breathwork (A): estimated fixed effects coefficient for etCO2 = -0.050, Std. Error = 0.008, df = 132.741, t = -5.7, p < 0.001; estimated fixed effects coefficient for Time = 0.106, Std. Error = 0.041, df = 159.437, t = 2.56, p = 0.011. Conscious-Connected breathwork (B): estimated fixed effects coefficient for etCO2 = -0.052, Std. Error = 0.009, df = 141.816, t = -5.7, p < 0.001; estimated fixed effects coefficient for Time = 0.184, Std. Error = 0.041, df = 158.137, t = 4.5, p < 0.001. Each box represents one measurement time point (1 to 6).

Supplementary References

Barsuglia, J., Davis, A. K., Palmer, R., Lancelotta, R., Windham-Herman, A.-M., Peterson, K., Polanco, M., Grant, R., & Griffiths, R. R. (2018). Intensity of Mystical Experiences Occasioned by 5-MeO-DMT and Comparison With a Prior Psilocybin Study. *Frontiers in Psychology*, *9*, 2459. https://doi.org/10.3389/fpsyg.2018.02459

Carbonaro, T. M., Johnson, M. W., Hurwitz, E., & Griffiths, R. R. (2018). Double-blind comparison of the two hallucinogens psilocybin and dextromethorphan: Similarities and differences in subjective experiences. *Psychopharmacology*, *235*(2), 521–534. https://doi.org/10.1007/s00213-017-4769-4

Carhart-Harris, R. L., Bolstridge, M., Day, C. M. J., Rucker, J., Watts, R., Erritzoe, D. E., Kaelen, M., Giribaldi, B., Bloomfield, M., Pilling, S., Rickard, J. A., Forbes, B., Feilding, A., Taylor, D., Curran, H. V., & Nutt, D. J. (2018). Psilocybin with psychological support for treatment-resistant depression: Six-month follow-up. *Psychopharmacology*, *235*(2), 399–408. https://doi.org/10.1007/s00213-017-4771-x

Carhart-Harris, R. L., Kaelen, M., Bolstridge, M., Williams, T. M., Williams, L. T., Underwood, R., Feilding, A., & Nutt, D. J. (2016). The paradoxical psychological effects of lysergic acid diethylamide (LSD). *Psychological Medicine*, *46*(7), 1379–1390. https://doi.org/10.1017/S0033291715002901

Griffiths, R. R., Johnson, M. W., Carducci, M. A., Umbricht, A., Richards, W. A., Richards, B. D., Cosimano, M. P., & Klinedinst, M. A. (2016). Psilocybin produces substantial and sustained decreases in depression and anxiety in patients with life-threatening cancer: A randomized double-blind trial. *Journal of Psychopharmacology (Oxford, England)*, *30*(12). https://doi.org/10.1177/0269881116675513

Holze, F., Vizeli, P., Müller, F., Ley, L., Duerig, R., Varghese, N., Eckert, A., Borgwardt, S., & Liechti, M. E. (2020). Distinct acute effects of LSD, MDMA, and D-amphetamine in healthy subjects. *Neuropsychopharmacology*, *45*(3), 462–471. https://doi.org/10.1038/s41386-019-0569-3

Holze, F., Vizeli, P., Ley, L., Müller, F., Dolder, P., Stocker, M., Duthaler, U., Varghese, N., Eckert, A., Borgwardt, S., & Liechti, M. E. (2021). Acute dose-dependent effects of lysergic acid diethylamide in a double-blind placebo-controlled study in healthy subjects. *Neuropsychopharmacology*, 46, 537–544; https://doi.org/10.1038/s41386-020-00883-6

Hysek, C. M., Simmler, L. D., Nicola, V. G., Vischer, N., Donzelli, M., Krähenbühl, S., Grouzmann, E., Huwyler, J., Hoener, M. C., & Liechti, M. E. (2012a). Duloxetine Inhibit Effects of MDMA ("Ecstacy") *In Vitro* and in Humans in a Randomized Placebo-Controlled Laboratory Study. *PLoS ONE 7(5): e36476. doi:10.1371/journal.pone.0036476*

Hysek, C. M., Brugger, R., Simmler, L. D., Bruggisser, M. N., Donzelli, M., Grouzmann, E., Hiener, M. C., & Liechti, M. E. (2012b). Clonidine and MDMA. *Journal of Pharmacology and Experimental Therapeutics*, *340* (2), 286-294. https://doi.org/10.1124/jpet.111.188425

Hysek, C. M., Schmid, Y., Rickli, A., & Liechti, M. E. (2013). Carvedilol inhibits the cardiostimulant and thermogenic effects of MDMA in humans: lost in translation. *British journal of pharmacology*, 170(6), 1273. https://doi.org/10.1111/bph.12398

Liechti, M.E., Dolder, P.C. & Schmid, Y. (2017). Alterations of consciousness and mystical-type experiences after acute LSD in humans. *Psychopharmacology* 234, 1499–1510. https://doi.org/10.1007/s00213-016-4453-0

Madsen, M. K., Fisher, P. M., Burmester, D., Dyssegaard, A., Stenbæk, D. S., Kristiansen, S., Johansen, S. S., Lehel, S., Linnet, K., Svarer, C., Erritzoe, D., Ozenne, B., & Knudsen, G. M. (2019). Psychedelic effects of psilocybin correlate with serotonin 2A receptor occupancy and plasma psilocin levels. *Neuropsychopharmacology*, *44*(7), Article 7. https://doi.org/10.1038/s41386-019-0324-9

Murray, C. H., Tare, I., Perry, C. M., Malina, M., Lee, R., & de Wit, H. (2022). Low doses of LSD reduce broadband oscillatory power and modulate event-related potentials in healthy adults. *Psychopharmacology*, *239*(6), 1735–1747. https://doi.org/10.1007/s00213-021-05991-9

Nicholas, C. R., Henriquez, K. M., Gassman, M. C., Cooper, K. M., Muller, D., Hetzel, S., Brown, R. T., Cozzi, N. V., Thomas, C., & Hutson, P. R. (2018). High dose psilocybin is associated with positive subjective effects in healthy volunteers. *Journal of Psychopharmacology (Oxford, England)*, *32*(7), 770–778. https://doi.org/10.1177/0269881118780713

Preller, K. H., Herdener, M., Pokorny, T., Planzer, A., Kraehenmann, R., Stämpfli, P., Liechti, M. E., Seifritz, E., & Vollenweider, F. X. (2017). The fabric of meaning and subjective effects in LSD-induced states depend on serotonin 2A receptor activation. Current Biology, 27(3), 451-457. https://doi.org/10.1016/j.cub.2016.12.030

Schmidt, A., Bachmann, R., Kometer, M., Csomor, P. A., Stephan, K. E., Seifritz, E., & Vollenweider, F. X. (2012). Mismatch Negativity Encoding of Prediction Errors Predicts S-ketamine-Induced Cognitive Impairments. *Neuropsychopharmacology*, *37*(4), Article 4. https://doi.org/10.1038/npp.2011.261

Smigielski, L., Kometer, M., Scheidegger, M., Krähenmann, R., Huber, T., & Vollenweider, F. X. (2019). Characterization and prediction of acute and sustained response to psychedelic psilocybin in a mindfulness group retreat. *Scientific Reports*, *9*(1), Article 1. https://doi.org/10.1038/s41598-019-50612-3

Smigielski, L., Kometer, M., Scheidegger, M., Stress, C., Preller, K. H., Koenig, T., & Vollenweider, F. X. (2020). P300-mediated modulations in self–other processing under psychedelic psilocybin are related to connectedness and changed meaning: A window into the self–other overlap. *Human Brain Mapping*, *41*(17), 4982–4996. https://doi.org/10.1002/hbm.25174

Wießner, I., Falchi, M., Palhano-Fontes, F., Feilding, A., Ribeiro, S., & Tófoli, L. F. (2023). LSD, madness and healing: Mystical experiences as possible link between psychosis model and therapy model. *Psychological Medicine*, *53*(4), 1151–1165. https://doi.org/10.1017/S0033291721002531
