# Supplementary material for: Decreased CO2 saturation during circular breathwork supports emergence of altered states of consciousness
Source: Commun Psychol. 2025 Apr 13;3:59. doi: 10.1038/s44271-025-00247-0 (PMC11994804; doi:10.1038/s44271-025-00247-0)
Supplement: Supplementary file 3 — Reporting Summary [file 44271_2025_247_MOESM3_ESM.pdf]

nature portfolio

Corresponding author(s): Martha N. Havenith

Last updated by author(s): Feb 4, 2025

Reporting Summary

Nature Portfolio wishes to improve the reproducibility of the work that we publish. This form provides structure for consistency and transparency in reporting. For further information on Nature Portfolio policies, see our Editorial Policies and the Editorial Policy Checklist.

Statistics

For all statistical analyses, confirm that the following items are present in the figure legend, table legend, main text, or Methods section.

n/a	Confirmed	
☐	☒	The exact sample size (*n*) for each experimental group/condition, given as a discrete number and unit of measurement
☐	☒	A statement on whether measurements were taken from distinct samples or whether the same sample was measured repeatedly
☐	☒	The statistical test(s) used AND whether they are one- or two-sided<br>*Only common tests should be described solely by name; describe more complex techniques in the Methods section.*
☒	☐	A description of all covariates tested
☐	☒	A description of any assumptions or corrections, such as tests of normality and adjustment for multiple comparisons
☐	☒	A full description of the statistical parameters including central tendency (e.g. means) or other basic estimates (e.g. regression coefficient) AND variation (e.g. standard deviation) or associated estimates of uncertainty (e.g. confidence intervals)
☐	☒	For null hypothesis testing, the test statistic (e.g. *F*, *t*, *r*) with confidence intervals, effect sizes, degrees of freedom and *P* value noted<br>*Give P values as exact values whenever suitable.*
☒	☐	For Bayesian analysis, information on the choice of priors and Markov chain Monte Carlo settings
☒	☐	For hierarchical and complex designs, identification of the appropriate level for tests and full reporting of outcomes
☐	☒	Estimates of effect sizes (e.g. Cohen's *d*, Pearson's *r*), indicating how they were calculated

Our web collection on statistics for biologists contains articles on many of the points above.

Software and code

Policy information about availability of computer code

Data collection	Data were entered manually in a Microsoft Excel sheet by two independent persons to ensure correct transcription from the paper forms that were completed during initial data collection.
Data analysis	Data were analysed using custom code written in Matlab (2019) and R (Version4.2.3).

For manuscripts utilizing custom algorithms or software that are central to the research but not yet described in published literature, software must be made available to editors and reviewers. We strongly encourage code deposition in a community repository (e.g. GitHub). See the Nature Portfolio guidelines for submitting code & software for further information.

Data

Policy information about availability of data

All manuscripts must include a data availability statement. This statement should provide the following information, where applicable:

- Accession codes, unique identifiers, or web links for publicly available datasets
- A description of any restrictions on data availability
- For clinical datasets or third party data, please ensure that the statement adheres to our policy

All anonymised data collected as well as all custom-written analysis code are available in the following repository: https://github.com/zero-noise-lab

Human research participants

Policy information about studies involving human research participants and Sex and Gender in Research.

Reporting on sex and gender	We recorded the self-reported gender of all participants. There were no significant differences in the gender composition of the experimental groups. Tests regarding gender-based differences in the effect of breathwork are shown in Supplementary Materials.
Population characteristics	In addition to self-reported gender, we recorded age and employment level of participants. These characteristics are summarized in the Methods section and detailed in Supplementary Materials. Participants were assigned to experimental conditions in a randomized rather than counterbalanced way, but none of the population characteristics differed significantly between experimental groups.
Recruitment	Since our study included experienced breathwork practitioners (mininum of 5 sessions completed prior to study), we recruited participants in a targeted way by posting study advertisements on the web page of the MIND Foundation, as well as in online communities of breathwork practitioners (via popular social media channels). As discussed in the manuscript, the fact that the participants had repeatedly done breathwork before makes it likely that they had positive expectations regarding its effects on their well-being.
Ethics oversight	Ethik-Kommission der Ärztekammer Berlin (Germany)

Note that full information on the approval of the study protocol must also be provided in the manuscript.

Field-specific reporting

Please select the one below that is the best fit for your research. If you are not sure, read the appropriate sections before making your selection.

☒ Life sciences ☐ Behavioural & social sciences ☐ Ecological, evolutionary & environmental sciences

For a reference copy of the document with all sections, see nature.com/documents/nr-reporting-summary-flat.pdf

Life sciences study design

All studies must disclose on these points even when the disclosure is negative.

Sample size	Given that research on breathwork is still scarce, we could not rely on previously reported effect sizes to estimate sample sizes directly. Instead, we used typical sample sizes in psychedelic studies as a reference, given the hypothesis that the evoked effects might be similar.
Data exclusions	Data regarding psychological follow-on effects were excluded if participants did not complete questionnaires for both measurement time points (1 week pre- and post-session). This criterion excluded data from 7 subjects for the Warwick-Edinburgh Mental Wellbeing Scale and 4 subjects for the Quick Inventory of Depressive Symptoms.
Replication	We have made all raw data and custom-written analysis codes available in a data repository (https://github.com/zero-noise-lab) so that they can be replicated. Two authors conducted statistical testing independently of each other to verify results.
Randomization	As mentioned in the manuscript, participants could freely choose between the two breathwork formats to ensure that they used the breathwork format they were most comfortable with. This should ensure that participants are proficient in the breathwork style they are asked to perform during the session. It also means that in both formats, participants were equally invested in the specific breathing technique they were using. Assignment to the passive or active breath group was randomized by handing participants sealed envelopes.
Blinding	Blinding was not strictly possible in this case, because both experimental factors - breathwork format was impossible to conceal from the facilitators and experimenters, and active versus passive breathing - while not disclosed to facilitators and experimenters - was easily visible from subjects' breathing rhythm. Since both CO2 and experience depth required documentation only, with no subjective ratings or input from experimenters, we believe that this non-blinded design was not detrimental to the study.

Reporting for specific materials, systems and methods

We require information from authors about some types of materials, experimental systems and methods used in many studies. Here, indicate whether each material, system or method listed is relevant to your study. If you are not sure if a list item applies to your research, read the appropriate section before selecting a response.

Materials & experimental systems

n/a	Involved in the study
☒	☐ Antibodies
☒	☐ Eukaryotic cell lines
☒	☐ Palaeontology and archaeology
☒	☐ Animals and other organisms
☒	☐ Clinical data
☒	☐ Dual use research of concern

Methods

n/a	Involved in the study
☒	☐ ChIP-seq
☒	☐ Flow cytometry
☒	☐ MRI-based neuroimaging
