# Supplementary material for: Decreased CO2 saturation during circular breathwork supports emergence of altered states of consciousness
Source: Commun Psychol. 2025 Apr 13;3:59. doi: 10.1038/s44271-025-00247-0 (PMC11994804; doi:10.1038/s44271-025-00247-0)
Supplement: Supplementary file 2 — Transparent Peer Review file [file 44271_2025_247_MOESM2_ESM.pdf]

nature portfolio

Peer Review File

Decreased CO2 saturation during circular breathwork supports emergence of altered states of consciousness

Corresponding Author: Dr Martha Havenith

This file contains all editorial decision letters in order by version, followed by all author rebuttals in order by version.

Version 0:

Decision Letter:

** Please ensure you delete the link to your author homepage in this e-mail if you wish to forward it to your coauthors **

Dear Dr Havenith,

Thank you for your patience during the peer-review process. Your manuscript titled "Decreased CO2 saturation during circular breathwork supports emergence of altered states of consciousness" has now been seen by 3 reviewers, whose comments are appended below. You will see that they find your work of some potential interest. However, they have raised some concerns that must be addressed. In light of these comments, we cannot accept the manuscript for publication, but would be interested in considering a revised version that fully addresses these concerns.

We hope you will find the Reviewers' comments useful as you decide how to proceed. Should additional work allow you to address these criticisms, we would be happy to look at a substantially revised manuscript. If you choose to take up this option, please highlight all changes in the manuscript text file, and provide a detailed point-by-point reply to the reviewers.

Please bear in mind that we will be reluctant to approach the reviewers again in the absence of substantial revisions.

Editorially, we consider it necessary that you address the comparability of the effects of circular breathwork to psychedelics and the appropriate tempering of conclusions. Regarding reviewer comments on hypotheses and preregistration, any a priori hypotheses should be reported, however we would not consider a retrospective registration of them at this point in time to contribute any added value. Moreover, the presence or absence of preregistration must be stated in the Methods. We would also like to emphasise that effect sizes for all statistical tests should be reported. We also encourage you to consider additional analytical approaches as suggested by Reviewer #2 that may be better suited to the data.

I am attaching a checklist that details critical reporting requirements for the revised manuscript. Please attend to each item and ensure your manuscript is fully compliant. We are requesting that your manuscript aligns with these requirements as this facilitates the evaluation of your manuscript, reducing delays in re-review and potential future acceptance. If your revised manuscript is not aligned with these requests on major issues, such as those concerning statistics, it may be returned to you for further revisions without re-review. Additional information can be found in our style and formatting guide Communications Psychology formatting guide.

If the revision process takes significantly longer than five months, we will be happy to reconsider your paper at a later date, provided it still presents a significant contribution to the literature at that stage.

We would appreciate it if you could keep us informed about an estimated timescale for resubmission, to facilitate our planning.

We are committed to providing a fair and constructive peer-review process. Please do not hesitate to contact us if you wish to discuss the revision in more detail.

Please use the following link to submit your
- revised manuscript,
- point-by-point response to the referees’ comments,

- cover letter (as a separate document),
- the Editorial Policy Checklist (see below),
- the Reporting Summary (see below), and
- the completed Editorial Request Table (attached):

Link Redacted

** This url links to your confidential home page and associated information about manuscripts you may have submitted or be reviewing for us. If you wish to forward this email to co-authors, please delete the link to your homepage first **

Thank you for the opportunity to review your work.

Best regards,

Daniel Quintana

Daniel Quintana, PhD
Editorial Board Member
Communications Psychology
orcid.org/0000-0003-2876-0004

REVIEWER EXPERTISE:
Reviewer #1 Cardiac/respiratory psychophysiology and psychedelics/altered consciousness
Reviewer #2 Cardiac/respiratory psychophysiology
Reviewer #3 Cardiac/respiratory psychophysiology

REVIEWER REPORTS:

Reviewer #1 (Remarks to the Author):

Overall, this is a much needed study in an area of interest to not only the scientific community but general public. I hope all my points raised here (some of which may not be accurate and need correcting) can aid in the iteration of this paper and improve the final manuscript in its published form. There are also opportunities to streamline the paper and make it more succinct/concise and thus more readable to the general public as well as researchers. In my opinion, the Results section should be shortened significantly with a great deal of content from this moved into the Methods section (which could be presented first – and thus no need to repeatedly state 'see Methods') and primarily the Discussion section. Nonetheless, this has very high potential for impact, and I welcome the authors to address and/or clarify the points raised below.

This is an amazing research effort by the author team, and has great potential to enhance and influence the literature on psychedelics (particularly nondrug compound alternatives), consciousness and interventions for potentially improving wellbeing. However, it is still important to note and empathise caution around the conclusions regarding effectiveness that can be drawn from a study that is not a pre-registered randomised placebo-controlled trial. The authors may consider retrospectively registering the study. This exploratory study marks an important step in breathwork research and moving the needle in how best behavioural intervention research like this can be conducted.

Abstract
Perhaps presenting some of the key quantitative data in the Abstract section would improve its impact.

-Should Holotropic Breathwork® be referred to as Grof® Breathwork now?

Introduction
Good, interesting scene-setting in the Introduction section but provide a table displaying the commonalities and differences between the two breathwork techniques so as to educate the reader visually as well.

Methods
Regarding structure, I'm aware that many journals present Results first, but always good to consider whether it aids telling the story of the current research study by presenting the Methods first. Personally, I read the Methods first so I had full context before reading the Results.

-Mention the year and date(s) the study was conducted.

-Mention if power calculation was used for recruiting sample.

-Is the passive breath group essentially a control? Or they were given an envelope telling them to breathe normally and therefore this was done to kind-of blind facilitators? I'm a little confused, sorry! :)

-Were the passive and active breath groups conducted separately, or were they in the same breathwork session? If they were in the same session, what is the impact of some participants essentially hyperventilating and others breathing normally and therefore being aware of their group allocation? Also, if the active and passive breath groups were taught in unison, I'm a little confused as to how this worked in terms of facilitator guidance? I could just not be understanding this correctly, so please make it clear. Were participants just told to ignore the breath-specific instructions from the breathwork teachers/facilitators?

-Mention if anything akin to a protocol pre-registration and statistical analysis plan were used. Are you able to retrospectively register this study?

-Any missing data? If so, any methods used to impute it?

-Provide reasoning for why only psilocibin was examined (was it due to prior literature(?) – e.g. Bahi et al.). Could you include comparisons to other classic psychedelics (LSD, DMT and mescaline)?

Results
Again I appreciate journals may have different requirements, but the Results read more like a Discussion. You can move much of the content/material to the Discussion (and some to the Methods), and this will dramatically shorten an otherwise very-lengthy Results section.

-The results section can be broken down using subheadings for the outcomes.

-If you reverse the structure (Methods before Results), you will not have to repeat certain things in this section. Again this will make the paper more concise and succinct, and the Results will be significantly shortened.

-Again (sorry!), point from Methods: When you say participants in the passive-breath group were instructed to adhere to their normal breathing rhythm, who instructed them to do this? The study coordinator via the envelopes or breathwork facilitators? Again I'm just slightly confused how this works if a facilitator is advising all participants to essentially hyperventilate – were passive-breath participants just told to ignore these breath-specific instructions? Just want this to be crystal clear to readers.

-State in methods that etCO2 was averaged from timepoints 2-4.

-Any particular reason why Dunn-Sidak was chosen over Bonferroni correction?

-I think you can move any dialogue and/or interpretation surrounding the results to the Discussion (again same for moving anything Methods related)

-You can provide full results of the ANOVAs, and include effect sizes (i.e., partial eta squared and/or Cohen's D).

-Several P values are zero. Do you mean "$p < 0.001$"?

-Also where P values are given as "$p < 0.002$" or "$p < 0.004$" do you mean "=" instead of "<" ?

-Why M±SEM instead of M±SD?

-Again, any discussion of results needs to be moved to Discussion section.

-May be due to journal requirements but, if not, feel free to move the Figures to where the results pertaining to said Figures are being reported.

-'Social contamination' – I like it! Move to Discussion.

-Move reference to statistical power to Methods and Discussion.

-Present effect sizes of t-tests, i.e., Cohen's D.

-Again, consistently check whether "<" is meant to be "=".

Discussion
Overall this reads well with great points made around set and setting, although, again, I believe most of your Results section can be presented in this Discussion section.

-Break this down into sections, including a 1) summary of the results, 2) the results in context along with 3) strengths, 4) limitations (the ones already discussed in the manuscript but also the ones mentioned from my peer-review which you agree

with) and future directions for research.

-Interesting to find similar effects of 1.5hr vs 3hr high-ventilation breathwork. Does this study finding tentatively posit that Consciously-Connected breathwork is perhaps slightly more ‘accessible’ then Holotropic?

- I appreciate how difficult it is to conduct such a study but there is still a chance that interruptions to the participants in order to collect etCO2 and subjective experience could have disrupted their experiences. This is a point for limitations – but could also be viewed in the opposite direction as they still had these ASC experiences even with the interruptions. Nuance needed.

-Would be great to hear authors’ key recommendations going forwards: What they have learnt and would advise on how to better collect these subjective experiences in particular in future studies.

Some more general notes include:
-Double-checking spelling/grammar and ensuring citations are consistent throughout the manuscript.

-Consider revising the Conflict of Interest statement if there are breathwork facilitators who are also authors on the paper. This is the first section I check before reading a paper.

Overall, amazing work. Thanks for the opportunity to read and review this manuscript. I hope these comments are constructive to improving the final paper.

Reviewer #2 (Remarks to the Author):

Summary: This is an interesting manuscript detailing the results of a randomized mechanistic study on physiological and psychological constructs implicated in altered states of consciousness produced during circular breathwork. The study advances our biopsychosocial understanding of how nonpharmacologically-i nduced ASCs improve mental health.

The issues that require being addressed are:

- Introduction
  - Consider replacing “consistently” with “consistent” throughout.
  - Include statement on type of study (e.g., exploratory mechanistic study”).
  - Include directional or non-directional hypotheses at the end of the introduction.
  - Consider removing statement of results from the last paragraph of the introduction.
- Results
  - Subscales of the 11-DASC were correlated with IL-1b change. Only 2 were statistically significant, and “Visual reconstruction” was not (p=.35) which should be mentioned more clearly.
  - p = 0 should be p < .001; all p and other statistics should be italicized, and p should be to 3 decimal places. Consider doing for other statistics as well.
  - There is quite a bit of interpretation in the results section. Consider saving any reflections/interpretations of the meaning of results for the discussion section.
  - There is quite a bit of visual interpretation of results. See comments below re methods section for application of more advanced statistics to test hypotheses.
  - Some methods statements appear to be repeated in the results section.
  - Fig 1.C and Fig 2.E x-axis should be scaled to actual minutes from baseline to accurately portray change.
  - Fig2.A and Fig2.B consider grouped bar chart for greater clarity.
  - Fig3.A and Fig3.B are somewhat uninterpretable. Consider other display options.
  - Fig3.C and Fig3.D are also somewhat uninterpretable. Figs also suggest that the analyses. Some of the colour coding used in Fig 3 C and D are difficult to differentiate. Suggest using a coding that does not relies on grayscale, not colour.
  - may not meet statistical assumptions. Consider natural logarithm of biomarkers.
  - All Fig 3 plots may be best approached with a mixed model derived trajectory.
- Discussion
  - Authors state that “...the experimental effects of circular breathwork are comparable to those evoked by psychedelics”. However, as shown in Figs 2A many elements of the 11-DASC did not reach the same level of intensity as psilocybin. The statement can only be plausibly made if the participants came from the same population. Since the experiment was done with experienced practitioners, who are likely to have more intense experiences and perceptions of the effects of breathwork than naïve populations, the results represent the upper limit of what can be expected from breathwork.
  - The concept of Awe suddenly appears in the final few sentences of the Discussion. This topic seems orthogonal to the rest of the paper, and it is not clear how this concept is related. More discussion is needed.
  - I appreciate the discussion on possible placebo/contextual factors that may play a role in inducing ASCs and improvements in depression and wellbeing. The authors may also consider discussing corrective emotional experiences as a common/contextual factor that may result from ASCs.
  - Although decreased etCO2 is correlated with deeper ASCs, it is not necessarily causal. Suggest tempering such conclusions. Given that participants have experience with breathwork practices, the first-person experience hyperventilation may be a trigger for inducing a placebo response of ASCs. That is, there may be a belief among experienced practitioners

that the more intense that the hyperventilation/breathwork is, the more profound their ASCs will be. This suggestion and belief are embedded within the ritual and so the placebo response cannot be ruled out. I would suggest expanding on this possibility based on
https://doi.org/10.1177/0269881116677852 and https://doi.org/10.3389/fpsyt.2019.00456
○ Consider discussion on limitations based on https://doi.org/10.1177/20451253231198466
• Methods
○ During the 1-week follow-up, were participants asked to abstain from breathwork?
○ Although these were experienced practitioners, were they asked about adverse effects, and if so, what was found?
○ Given that rich temporal data were collected, consider using mixed modeling approach to address hypotheses.
○ Consider using multivariate mixed modeling approach rather than visual inspection for temporal co-regulation.
○ Salivary biomarkers may need a natural logarithm transformation to meet assumptions of statistical tests.
○ Need include statement of meeting statistical assumptions in methods or results section.
○ Consider sharing code and output from MATLAB and R.

Reviewer #3 (Remarks to the Author):

I think this is an idea that people need to hear. I think that people sometimes assume that you get what you pay for (i.e., psychotropic drugs are more expensive and have more side effects so they must have stronger positive effects), but sometimes that is not true, and this appears to be one of those times.

Impressive amount of measures. Unfortunate that there were not more participants.

Please add effect sizes, ideally Cohen's d, even when effects are not significant. The effect size can still be informative.

I wish that the two conditions could have been the same length, but maybe that was impossible because there are clearly set session lengths for these two types of breathing exercises.

It would have been better if there had been another score for when participants could not respond instead of giving them the highest score in the altered states of consciousness scale. I think actively saying you are a 5 on that scale and not responding to the question are not necessarily the same thing. I do not see this as a fatal flaw because the authors also have the physiological measures.

EDITORIAL POLICIES

We ask that you ensure your manuscript complies with our editorial policies and reporting requirements.

To that end, we require revised manuscripts to be accompanied by two completed items: a reporting summary that collects information on study design and procedure, and an editorial policy checklist that verifies compliance with all required editorial policies

Nature Research Reporting Summary

• Editorial Policy Checkl ist

All points on the policy checklist must be addressed. Your revised manuscript can only be sent back to the referees if these checklists are completed and uploaded with the revision.

Notes: If you have submitted a Stage 1 Registered Report, Review, Primer, Comment, or Perspective you do not need to submit these forms. If you have already submitted these forms, you may disregard this request.

* TRANSPARENT PEER REVIEW: Communications Psychology uses a transparent peer review system. This means that we publish the editorial decision letters including Reviewers' comments to the authors and the author rebuttal letters online as a supplementary peer review file. However, on author request, confidential information and data can be removed from the published reviewer reports and rebuttal letters prior to publication. If your manuscript has been previously reviewed at another journal, those Reviewers' comments would not form part of the published peer review file.

** Visit Nature Research's author and referees' website at www.nature.com/authors for information about policies, services and author benefits**

Communications Psychology is committed to improving transparency in authorship. As part of our efforts in this direction, we are now requesting that all authors identified as 'corresponding author' create and link their Open Researcher and Contributor Identifier (ORCID) with their account on the Manuscript Tracking System prior to acceptance. ORCID helps the scientific community achieve unambiguous attribution of all scholarly contributions. You can create and link your ORCID from the home page of the Manuscript Tracking System by clicking on 'Modify my Springer Nature account' and following the instructions in the link below. Please also inform all co-authors that they can add their ORCIDs to their accounts and that they must do so prior to acceptance.
https://www.springernature.com/gp/researchers/orcid/orcid-for-nature-research

For more information please visit http://www.springernature.com/orcid

If you experience problems in linking your ORCID, please contact the Platform Support Helpdesk.

Version 1:

Decision Letter:

** Please ensure you delete the link to your author homepage in this e-mail if you wish to forward it to your coauthors **

Dear Dr Havenith,

Your manuscript titled "Decreased CO2 saturation during circular breathwork supports emergence of altered states of consciousness" has now been seen by our reviewers, whose comments appear below. In light of their advice I am delighted to say that we are happy, in principle, to publish a suitably revised version in Communications Psychology.

We therefore invite you to revise your paper one last time to address the remaining concerns of our reviewers and a list of editorial requests. At the same time we ask that you edit your manuscript to comply with our format requirements and to maximise the accessibility and therefore the impact of your work.

EDITORIAL REQUESTS:

Please review our specific editorial comments and requests regarding your manuscript in the attached "Editorial Requests Table". Please outline your response to each request in the right hand column. Please upload the completed table with your manuscript files as a Related Manuscript file.

If you have any questions or concerns about any of our requests, please do not hesitate to contact me.

SUBMISSION INFORMATION:

In order to accept your paper, we require the files listed here https://www.nature.com/documents/commsj-file-checklist.pdf .

OPEN ACCESS:

Communications Psychology is a fully open access journal. Articles are made freely accessible on publication. For further information about article processing charges, open access funding, and advice and support from Nature Research, please visit https://www.nature.com/commspsychol/open-access

At acceptance, you will be provided with instructions for completing the open access licence agreement on behalf of all authors. This grants us the necessary permissions to publish your paper. Additionally, you will be asked to declare that all required third party permissions have been obtained, and to provide billing information in order to pay the article-processing charge (APC).

* TRANSPARENT PEER REVIEW: Communications Psychology uses a transparent peer review system. On author request, confidential information and data can be removed from the published reviewer reports and rebuttal letters prior to publication. If you are concerned about the release of confidential data, please let us know specifically what information you would like to have removed. Please note that we cannot incorporate redactions for any other reasons.

* CODE AVAILABILITY: All Communications Psychology manuscripts must include a section titled "Code Availability" at the end of the methods section. We require that the custom analysis code supporting your conclusions is made available in a publicly accessible repository at this stage; please choose a repository that generates a digital object identifier (DOI) for the code; the link to the repository and the DOI must be included in the Code Availability statement. Publication as Supplementary Information will not suffice.

* DATA AVAILABILITY:
All Communications Psychology manuscripts must include a section titled "Data Availability" at the end of the Methods section. More information on this policy, is available in the Editorial Requests Table and at <a

href="http://www.nature.com/authors/policies/data/data-avai lability-statements-data-citations.pdf">http://www.nature.com/authors/policies/data/data-avai labil ity-statements-data-citations.pdf.

Please use the following link to submit the above items:
Link Redacted
** This url links to your confidential home page and associated information about manuscripts you may have submitted or be reviewing for us. If you wish to forward this email to co-authors, please delete the link to your homepage first **

We hope to hear from you within two weeks; please let us know if you need more time.

Best regards,

Jennifer Bellingtier

Jennifer Bellingtier, PhD
Senior Editor
Communications Psychology

Daniel Quintana, PhD
Editorial Board Member
Communications Psychology
orcid.org/0000-0003-2876-0004

REVIEWERS' EXPERTISE:

Reviewer #1 Cardiac/respiratory psychophysiology and psychedelics/altered consciousness
Reviewer #2 Cardiac/respiratory psychophysiology

REVIEWERS' COMMENTS:

Reviewer #1 (Remarks to the Author):

Concerns have now been addressed. Great job by the Authors and the Editor. Cheers to the other Reviewers as well.

Reviewer #2 (Remarks to the Author):

The authors did a thorough job in responding to my earlier comments. I have no further comments.

** Visit Nature Research's author and referees' website at www.nature.com/authors for information about policies, services and author benefits**

Open Access This Peer Review File is licensed under a Creative Commons Attribution 4.0 International License, which permits use, sharing, adaptation, distribution and reproduction in any medium or format, as long as you give appropriate credit to the original author(s) and the source, provide a link to the Creative Commons license, and indicate if changes were made.
In cases where reviewers are anonymous, credit should be given to 'Anonymous Referee' and the source.
The images or other third party material in this Peer Review File are included in the article's Creative Commons license, unless indicated otherwise in a credit line to the material. If material is not included in the article's Creative Commons license and your intended use is not permitted by statutory regulation or exceeds the permitted use, you will need to obtain permission directly from the copyright holder.
To view a copy of this license, visit https://creativecommons.org/licenses/by/4.0/

Point-by-point reply "Decreased CO2 saturation during circular breathwork supports emergence of altered states of consciousness"

We thank the editor and reviewers for their extremely helpful comments and suggestions. Based on their feedback, we have substantially revised the manuscript. This includes adding a mixed-model analysis of the relations between partial CO_2 pressure ($etCO_2$) and subjective experience, transforming all biomarker measurements using their natural logarithm, adding effect sizes for all statistical tests, and adding benchmark 11-DASC and MEQ-30 scores for two additional psychedelic substances (LSD and MDMA) to compare to the subjective effects of breathwork, as well as a table comparing attributes of the two breathwork styles we tested. We have also adjusted our conclusions regarding the comparability of breathwork to psychedelic experiences, added an extended discussion of potential caveats and limitations of our study, as well as suggestions for future directions of research. Finally, we have restructured the manuscript to more clearly delineate methods, results and discussion (and their sub-sections), and to clarify the design of our experimental intervention

REVIEWER REPORTS

Reviewer #1

Overall, this is a much needed study in an area of interest to not only the scientific community but general public. I hope all my points raised here (some of which may not be accurate and need correcting) can aid in the iteration of this paper and improve the final manuscript in its published form. There are also opportunities to **streamline the paper** and make it more succinct/concise and thus more readable to the general public as well as researchers. In my opinion, the **Results section should be shortened** significantly with a great deal of **content from this moved into the Methods section** (which could be presented first – and thus no need to repeatedly state 'see Methods') **and primarily the Discussion section**. Nonetheless, this has very high potential for impact, and I welcome the authors to address and/or clarify the points raised below.

This is an amazing research effort by the author team, and has great potential to enhance and influence the literature on psychedelics (particularly nondrug compound alternatives), consciousness and interventions for potentially improving wellbeing. However, it is still important to note and empathise **caution around the conclusions regarding effectiveness** that can be drawn from a study that is not a preregistered randomised placebo-controlled trial. The authors may consider retrospectively registering the study. This **exploratory study** marks an important step in breathwork research and moving the needle in how best behavioural intervention research like this can be conducted.

We thank Reviewer 1 for their kind and very helpful feedback. As suggested, we have exchanged the order of Methods and Results section, moved technical information from Results to Methods, and more interpretative sections from Results to Discussion (see below).

Abstract
Perhaps presenting some of the **key quantitative data** in the Abstract section would improve its impact.
We thank the reviewer for this suggestion, and have added the correlation coefficient and statistical significance for one central finding (the relation between CO_2 and experience depth) in the abstract. Other results relied on more complex quantitative data (e.g. multiple correlations) that in our view would have compromised the length/readability of the abstract.

-Should Holotropic Breathwork® be referred to as Grof® Breathwork now?

Thank you for pointing this out. After conferring with the facilitators invovled, the most accurate description now appears to be holotropic breathwork without the trademark. We have updated this across the manuscript.

Introduction

Good, interesting scene-setting in the Introduction section but provide a table displaying the commonalities and differences between the two breathwork techniques so as to educate the reader visually as well.

We thank the reviewer for this very helpful suggestion and have added the new Table 1 accordingly.

Methods

Regarding structure, I'm aware that many journals present Results first, but always good to consider whether it aids telling the story of the current research study by presenting the **Methods first**. Personally, I read the Methods first so I had full context before reading the Results.

We agree, and have changed the manuscript structure to present Methods before Results.

-Mention the **year and date(s) the study was conducted**.

We have added this information in the 'Subjects and Ethics' section of Methods.

-Mention **if power calculation was used for recruiting sample**.

This study was largely exploratory, and since we could only rely on an extremely small pool of previous literature regarding the physiological and psychological effects of circular breathwork. As such, we estimated the size of the recruiting sample by considering standard psychedelic studies, which typically feature sample sizes of 15-25 participants, and assuming that the effects of breathwork were like to be at least somewhat less pronounced, bringing us to a conservative sample size of n = 36. We have now added this information in the section 'Subjects and Ethics' of Methods.

-Is the passive breath group essentially a control? Or they were given an envelope telling them to breathe normally and therefore this was done to kind-of blind facilitators? I'm a little confused, sorry! :)

-**Were the passive and active breath groups conducted separately, or were they in the same breathwork session**? If they were in the same session, what is the impact of some participants essentially hyperventilating and others breathing normally and therefore being aware of their group allocation? Also, if the active and passive breath groups were taught in unison, I'm a little confused as to how this worked in terms of facilitator guidance? I could just not be understanding this correctly, so please make it clear. Were participants just told to ignore the breath-specific instructions from the breathwork teachers/facilitators?

We thank the reviewer for highlighting these points of confusion and have clarified and expanded the descriptions of the experimental groups in the Methods and Results section. Active and passive breath participants indeed took part in the same sessions to equalize context factors like music, duration, pre-session sharing and 'social contagion' by other participants. Participants were indeed assigned their experimental group randomly (i.e. by lottery), and facilitators were pseudo-blinded to the participants' experimental condition, in the sense that they were not explicitly informed of it, but in many cases could probably derive it from the participant's breathing pattern. We now mention this information in the 'Interventions' section of Methods and summarize it briefly in the first paragraph of Results.

-Mention if anything akin to a protocol pre-registration and statistical analysis plan were used. Are you able to retrospectively register this study?

Unfortunately we cannot register our study in retrospect. What's more, given the distinct lack of scientific literature on the physiological and neuronal mechanisms of breathwork so far, our approach in this study was largely exploratory, in the sense that we had no directional hypotheses at all regarding the question if and how CO_2 saturation would predict experience depth. As such, our statistical analysis plan was to a) test differences in CO_2 saturation and experience depth between the two experimental groups (ANOVA) and b) if such differences were confirmed, to test the relation of changes in CO_2 saturation and experience depth over time (Correlation). However, this analysis plan was not registered. In the revised manuscript we have now improved statistical analyses using a mixed-model approach (see new Figures 3C and S3). To allow readers to explicitly take the exploratory nature and lack of pre-registration of this study into account in evaluating our results, we have now added this information in section 'Subjects and Ethics' section of Methods, and also highlight the exploratory nature of this study in the last paragraph of the Introduction and the first paragraph of the Discusssion.

-Any missing data? If so, any methods used to impute it?

Yes, there were indeed some missing questionnaire responses (n = 8 out of 33), as well as some inconclusive biomarker measurements (n= 6 out of 61). We did not use any imputing methods, instead we only included complete data sets in each analysis. As a result, the number of participants differs somewhat between the analyses shown in Figures 1-3, Figure 4 and Figure 5. We have now clarified this information in the sections 'Self-reports on mental health and well-being', 'Measurement of Alpha-amylase and IL-1Beta in saliva' and 'Data analysis' of Methods, and summarized the number of participants included in each analysis in the new Supplementary Table S2.

-Provide reasoning for why only psilocibin was examined (was it due to prior literature – e.g. Bahi et al.). Could you include comparisons to other classic psychedelics (LSD, DMT and mescaline)?

We thank the reviewer for this suggestion, and have now added LSD and MDMA as additional benchmark comparisons. We focused on these three substances, because for these the Altered States Database (see Prugger et al., 20222) we used to extract reference scores for this analysis yielded a sufficient number of studies that could be pooled. Specifically, we included substances for which at least three independent studies with comparable ingested dosages could be pooled together both for the 11-DASC and the MEQ-30. The added scores can be seen in the new Figures 2A,B and are also described in Supp. Tables S3 and S4. The explanation of our database search has been extended in section 'Data base analysis of MEQ30 and 11-DASC reference scores' of Methods.

Results

Again I appreciate journals may have different requirements, but the Results read more like a Discussion. You can move much of the content/material to the Discussion (and some to the Methods), and this will dramatically shorten an otherwise very-lengthy Results section.

We agree with the reviewer and have moved more interpretative texts to the Discussion (see e.g. paragraphs 3 and 5, Lines 814-822 and 845-858).

-The results section can be broken down using subheadings for the outcomes.

Following the reviewer's suggestion, we have broken down the Results section into the following five sub-headings:

End-tidal CO2 saturation during breathwork

Subjective experience depth during breathwork

Relationship between end-tidal CO2 and experience depth

Sustained psychological effects of breathwork

Subacute physiological effects of breathwork

-If you reverse the structure (Methods before Results), you will not have to repeat certain things in this section. Again this will make the paper more concise and succinct, and the Results will be significantly shortened.

We thank the reviewer for pointing this out, and have eliminated or shortened repeated information from the Results section (see e.g. first paragraph).

-Again (sorry!), point from Methods: When you say participants in the passive-breath group were instructed to adhere to their normal breathing rhythm, who instructed them to do this? The study coordinator via the envelopes or breathwork facilitators? Again I'm just slightly confused how this works if a facilitator is advising all participants to essentially hyperventilate – were passive-breath participants just told to ignore these breath-specific instructions? Just want this to be crystal clear to readers.

This is correct – Once participants had been assigned to the passive-breath group randomly, the study coordinator asked them to ignore the breathing instructions of the facilitators and instead adhere to their normal breathing rhythm. At the beginning of the session, facilitators also reminded particpants to only follow breathing instructions if they had been assigned to the active-breath group. We have now clarified this procedure in the section 'Interventions' of Methods and summarized it in the first paragraph of Results.

-State in methods that etCO2 was averaged from timepoints 2-4.

We have added this information in the 'Data Analysis' section of Methods.

-Any particular reason why Dunn-Sidak was chosen over Bonferroni correction?

The main reason for our choice was that the Bonferroni correction is thought to underestimate the statistical significance of multiple comparisons (e.g. Francis and Thunell 2021; Narum 2006; Mathur and VanderWeele 2018). Since the Dunn-Sidak correction is at least marginally less conservative and in some views better interpretable (e.g. Midway et al. 2020; Ludbrook 1991), we opted for this aproach.

-I think you can move any dialogue and/or interpretation surrounding the results to the Discussion (again same for moving anything Methods related)

Thank you, we have done so (see above for details).

-You can provide full results of the ANOVAs, and include effect sizes (i.e., partial eta squared and/or Cohen's D).

We have now added effect sizes (Cohen's D) both in the Results section (see e.g. lines 471-478) and in Supplementary Table S5.

-Several P values are zero. Do you mean "$p < 0.001$"?

Yes indeed (or '$p < 0.0000001$' in most cases), we have amended this throughout the text.

-Also where P values are given as "$p < 0.002$" or "$p < 0.004$" do you mean "=" instead of "<" ?

In these cases, we always gave the rounded-up number as an upper bound, since we did not want to underestimate p-values that had been rounded down. We have now changed all p-values to exact values, which are rounded to two decimals when they exceed $p = 0.01$ (e.g. $p = 0.03$), and to three decimals when they fall between $0 = 0.001$ and $p = 0.01$ (e.g. $p = 0.004$).

-Why M±SEM instead of M±SD?

We used the SEM in this context because the inference to the population mean seemed more relevant than the spread of values within the sample.

-Again, any discussion of results needs to be moved to Discussion section.

Thank you, we have done so (for details, please see above).

-May be due to journal requirements but, if not, feel free to move the Figures to where the results pertaining to said Figures are being reported.

We agree with the reviewer and have now placed the figures throughout the Results section.

-'Social contamination' – I like it! Move to Discussion.

Thank you, this argument now appears in the Discussion, subsection '*Subjective effects of breathwork*', paragraph 5.

-Move reference to statistical power to Methods and Discussion.

This is now mentioned in lines 888-893.

-Present effect sizes of t-tests, i.e., Cohen's D.

We have added effect sizes for all statistical tests, which are now presented throughout the main text as well as in Supplementary Table S5.

-Again, consistently check whether "<" is meant to be "=".

Thank you, we have adjusted all p-values accordingly (see above).

Discussion

Overall this reads well with great points made around set and setting, although, again, I believe most of your Results section can be presented in this Discussion section.

-Break this down into sections, including a 1) summary of the results, 2) the results in context along with 3) strengths, 4) limitations (the ones already discussed in the manuscript but also the ones mentioned from my peer-review which you agree with) and future directions for research.

We thank the reviewer for pointing this out and have restructured the Discussion to now begin with a summary, and then discuss results, context, limitations and future recommendations for each of the following (new) sub-sections:

Subjective effects of breathwork

Comparison between breathwork traditions

Sustained psychological effects of breathwork

Subacute physiological effects of breathwork

These sections also include the new arguments brought up throughout the review process.

-Interesting to find similar effects of 1.5hr vs 3hr high-ventilation breathwork. Does this study finding tentatively posit that Consciously-Connected breathwork is perhaps slightly more ‘accessible’ then Holotropic?

Yes, that is indeed one of the tentative conclusions of this study. We wanted to avoid stating conclusions that might be interpreted as overly prescriptive (e.g. ‘Consciously-Connected breathwork is more efficient than Holotropic breathwork!’). We do however agree with the reviewer that this is a valid interpretation of the data (especially since Holotropic breathwork also features paired sessions, so that each participant typically completes one session as a breather and one as a sitter, adding up to an approximately triple to quadruple time investment). We have now added a statement to this effect in Line 932-940 of the Discussion (subsection ‘*Comparison between breathwork styles*’).

- I appreciate how difficult it is to conduct such a study but there is still a chance that interruptions to the participants in order to collect etCO2 and subjective experience could have disrupted their experiences. This is a point for limitations – but could also be viewed in the opposite direction as they still had these ASC experiences even with the interruptions. Nuance needed.

We thank the reviewer for pointing this out, and have added these arguments to the Discussion (Lines 798-800 and 906-915, subsection ‘*Subjective effects of breathwork*’).

-Would be great to hear authors’ key recommendations going forwards: What they have learnt and would advise on how to better collect these subjective experiences in particular in future studies.

Thank you, we have added future recommendations to the discussion. In our view, the main interesting points of improvement are the following: In terms of ratings during the session, reviewer 3 suggested to assign separate scores for participants actively signalling a score of 5, and participants being unable to respond at all. We agree that this would give a better differentiation of subjective experiences throughout the session. In terms of post-session self-reports, we have so far used classical questionnaires developed mainly to assess psychedelic experiences, and while this is great in terms of direct comparisons with previous literature, it may also miss out on aspects that may be more unique to breathwork. As a complementary approach to these classical questionnaires, we would therefore aim to also collect free reports of experiences, and extract e.g. emotional content using deep learning tools in order to identify potential experiential dimensions that need to be considered in addition to those highlighted by psychedelic research. Beyond self-reports, we would also propose to directly derive experiential states from behavioural analyses (e.g. using video recordings during a breathwork session), which would also add improved temproal resolution – which in turn would allow for direct links to neuronal activity recorded simultaneously.

As the reviewer pointed out above, our measurements throughout the session do by definition disrupt subjective experiences to some degree. A useful additional metric would therefore also be to let participants rate the degree of disruption to their session that they experienced during measurements.

Finally, here we explore the influence of set and setting by keeping set and setting steady while varying breathing technique across experimental conditions. In a follow-up study, the complementary manipulation would be fascinating to explore – i.e. keeping breathing technique constant, but adding context factors such as music, group sharing and communal sessions in one experimental group, while giving individual sessions without music in the other.

These points have been added to lines 864-893 in the Discussion (paragraphs 5-6 of subsection '*Subjective effects of breathwork*').

Some more general notes include:

-Double-checking spelling/grammar and ensuring citations are consistent throughout the manuscript.

Thank you, we have done so.

-Consider revising the Conflict of Interest statement if there are breathwork facilitators who are also authors on the paper. This is the first section I check before reading a paper.

We thank the reviewer for pointing this out! Of the authors, Martha Havenith is trained as a Consciously-Connected breathwork facilitator, Max Leidenberger as a breathwork facilitator in the 'Lichtatmung' version of Consciously-Connected breathwork, and Simona Rakusa, Marijan Bernardic and Liliana Vasquez-Mock are trained as facilitators in Holotropic breathwork. We have added this information in the Conflict of Interest statement.

Overall, amazing work. Thanks for the opportunity to read and review this manuscript. I hope these comments are constructive to improving the final paper.

Thank you very much for the helpful and encouraging feedback!

Reviewer #2

Summary: This is an interesting manuscript detailing the results of a randomized mechanistic study on physiological and psychological constructs implicated in altered states of consciousness produced during circular breathwork. The study advances our biopsychosocial understanding of how nonpharmacologically-induced ASCs improve mental health.

The issues that require being addressed are:

- Introduction

○ Consider replacing “consistently” with “**consistent**” throughout.

Thank you, we have done so.

o Include statement on type of study (e.g., exploratory mechanistic study”).

We have now added this information in the last paragraph of the Introduction, the first paragraph of Methods, and the first pragraph of the Discussion.

○ Include directional or non-directional hypotheses at the end of the introduction.

Given the exploratory nature of this study, the only hypotheses that were directional were that CO_2 saturation would decrease during the session. All other hypotheses regarding subjective experiences, their relation to CO_2 saturation, as well as to follow-on effects, were non-directional. We now menton this in the last sentence of the introduction. In the same paragraph, we also highlight again that the current study is exploratory.

○ Consider removing statement of results from the last paragraph of the introduction. Thank you, we

have cut this paragraph and moved corresponding statements into Results and Discussion.

- Results

○ Subscales of the 11-DASC were correlated with IL-1b change. Only 2 were statistically significant, and "Visual reconstruction" was not (p=.35) which should be mentioned more clearly.

We thank the reviewer for pointing this out – there was in fact a typo in this p-value. It was p = 0.035, so that the correlation of all three sub-scales with IL-1b was significant. In the updated analysis, as per the reviewer's suggestion, we have LN-transformed biomarker values. Spearman rho and p values are correspondingly updated in the Results subsection '*Subacute physiological effects of breathwork',* and all are statistically significant (Lines 748-752).

○ p = 0 should be p < .001; all p and other statistics should be italicized, and p should be to 3 decimal places. Consider doing for other statistics as well.

Thank you, we have corrected this throughout the text, and are now showing p with 2 decimal places when p >= 0.01 and with three decimal places when p < 0.01 (see e.g. Line 619).

○ There is quite a bit of interpretation in the results section. Consider saving any reflections/interpretations of the meaning of results for the discussion section.

Thank you, we have moved more interpretative texts into the Discussion (see e.g. Lines 815-821 and 865-868).

○ There is quite a bit of visual interpretation of results. See comments below re methods section for application of more advanced statistics to test hypotheses.

We have amended this by adding a new mixed-model analysis based on the comments below.

○ Some methods statements appear to be repeated in the results section.

We have shortened such repeat statements to only include information that is crucial to understand the main results, and also moved the Methods section in front of Results to provide necessary information about out approach upfront.

○ Fig 1.C and Fig 2.E x-axis should be scaled to actual minutes from baseline to accurately portray change.

We unfortunately cannot change the axis labels in the suggested way, because we collected six measurement time points for both breathwork styles, but due to their different overall durations (Holotropic: approx. 3 hours, Consciously-connected: approx 90 minutes), measurements are taken approx. every 30 minutes during Holotropic breathwork sessions, but every 15 minutes during a

Consciously-connected breathwork session. We are now highlighting this difference directly in the figure legends of Figures 1C and 2E in order to make it more salient.

○ Fig2.A and Fig2.B consider grouped bar chart for greater clarity.

Thank you for this suggestion. Given that we have now also added benchmark scores for LSD and MDMA, the resulting bar charts would have contained too many distinct groups to be easily distinguishable visually. We therefore switched back to line plots with a new visual layout and colour palate to enhance clarity.

○ Fig3.A and Fig3.B are somewhat uninterpretable. Consider other display options.

We thank the reviewer for pointing this out and have replaced the corresponding plots with the new Figures 3A-B and S3. In these figures, data points are shown separately for the six measurement time points as well as the two breathwork styles, so that they should be easier to disentangle visually.

○ Fig3.C and Fig3.D are also somewhat uninterpretable. Figs also suggest that the analyses. Some of the colour coding used in Fig 3 C and D are difficult to differentiate. Suggest using a coding that relies on grayscale, not colour.

For better interpretability, early versus late measurement time points are now translated to luminance of the data points only, not to changes in color as the reveiwer suggested (see the new Figs. 3C-D).

○ may not meet statistical assumptions. Consider natural logarithm of biomarkers.

We thank the reviewer for pointing this out and have implemented this logarithmic transformation in the updated Figure 5. Please note that the corresponding statistical tests were not affected by the transformation since we used non-paremetric testing for our biomarker analyses.

○ All Fig 3 plots may be best approached with a mixed model derived trajectory.

We thank the reviewer for this suggestion, and have implemented this analysis, now represented in the new Figure 3 as well as Supp. Figure S3.

- Discussion

○ Authors state that "...the experimental effects of circular breathwork are comparable to those evoked by psychedelics". However, as shown in Figs 2A many elements of the 11-DASC did not reach the same

level of intensity as psilocybin. The statement can only be plausibly made if the participants came from the same population. Since the experiment was done with experienced practitioners, who are likely to have more intense experiences and perceptions of the effects of breathwork than naïve populations, the results represent the upper limit of what can be expected from breathwork.

We agree with the reviewer, and have rephrased the corresponding statements (see e.g. Lines 37-39 in the abstract, line 95 in the Introduction, and lines 508-509 and 517-538 of Results).

○ The concept of Awe suddenly appears in the final few sentences of the Discussion. This topic seems orthogonal to the rest of the paper, and it is not clear how this concept is related. More discussion is needed.

We have now extended the explanation of this point to clarify the conceptual relevance for readers (see Lines 997-1008, paragraph 5 of subsection '*Sustained psychological effects of breathwork*' in the Discussion). If this should still appear disjointed, we can remove it from the text.

○ I appreciate the discussion on possible placebo/contextual factors that may play a role in inducing ASCs and improvements in depression and wellbeing. The authors may also consider discussing corrective emotional experiences as a common/contextual factor that may result from ASCs.

We thank the reviewer for pointing this out and have added a mention of corrective emotional exeriences in breathwork to the Discussion (Lines 987-997; paragraph 4 of subsection '*Sustained psychological effects of breathwork*').

○ Although decreased etCO2 is correlated with deeper ASCs, it is not necessarily causal. Suggest tempering such conclusions. Given that participants have experience with breathwork practices, the first-person experience hyperventilation may be a trigger for inducing a placebo response of ASCs. That is, there may be a belief among experienced practitioners that the more intense that the hyperventilation/breathwork is, the more profound their ASCs will be. This suggestion and belief are embedded within the ritual and so the placebo response cannot be ruled out. I would suggest expanding on this possibility based on https://doi.org/10.1177/0269881116677852 and https://doi.org/10.3389/fpsyt.2019.00456

This is a very interesting point, which we now address in Lines 846-860 of the Discussion (subsection '*Subjective effects of breathwork*', paragraph 4). In this paragraph, we have also added a suggestion for future work to repeat similar measurements in naïve participants (Lines 856-860). This would simultaneously address the question whether deep subjective experiences still occur in the absence of intense breathing (as we observed in a few participants in this study), or if such 'CO2-independent' experiences only occur in experienced breathworkers.

○ Consider discussion on limitations based on https://doi.org/10.1177/20451253231198466

We thank the reviewer for this suggestion, and now address some of these potential limitations in Lines 851-853 as well as 896-916 aand 950-951. We do also believe that by including our passive-breath group, at least some common context effects are addressed. At the same time, we agree that potential beliefs involved in placebo effects as well as effects based on participant selection can definitely be addressed further in future studies (see Lines 896-916).

- Methods

○ During the 1-week follow-up, were participants asked to abstain from breathwork?

We did not ask participants to abstain from breathwork or similar activities, but we did ask participants to list any emotionally significant events, including additional self-development practices, that had taken place between the breathwork session and the time of filling out the follow-up questionnaires. According to the responses to this question, none of the participants had experienced further breathwork sessions prior to completing the 1-week follow-up survey. More specifically, according to the responses to this question, two participants engaged in daily meditation practices following the breathwork session, two started new romantic relationships, and one had a psychotherapy session prior to completing the one-week follow-up survey. We have added this information to Line 322-329 of Methods, subsection *Self-reports on mental health and well-being*.

○ Although these were experienced practitioners, were they asked about adverse effects, and if so, what was found?

In the current study we did not explicitly ask about adverse effects, but rather gave participants space for general feedback about the session. In this general feedback, no adverse effects were mentioned. We have added a suggestion to add a more explicit question on adverse effects in future studies in Lines 974-980 of the Discussion, subsection *Sustained psychological effects of breathwork*, paragraph 3.

○ Given that rich temporal data were collected, consider using mixed modeling approach to address hypotheses.
○ Consider using multivariate mixed modeling approach rather than visual inspection for temporal co-regulation.

We thank the reviewer for this suggestion, and have implemented this analysis (see the new Figure 3 as well as Supp. Figure S3).

○ Salivary biomarkers may need a natural logarithm transformation to meet assumptions of statistical tests.

We thank the reviewer for pointing this out and have implemented this transformation in the updated Figure 5. Please note that the corresponding statistical tests were not affected by the transformation since we used non-paremetric testing for our biomarker analyses.

○ Need to include statement of meeting statistical assumptions in methods or results section.

Thank you, we have added this information in Lines 378-425 of Methods, subsection *Statistics*.

o Consider sharing code and output from MATLAB and R.

We have now deposited the code for this paper in the following github repository: github.com/zeronoiselab/BreathworkCO2.

Reviewer #3

I think this is an idea that people need to hear. I think that people sometimes assume that you get what you pay for (i.e., psychotropic drugs are more expensive and have more side effects so they must have stronger positive effects), but sometimes that is not true, and this appears to be one of those times.

We would like to thank reviewer 3 for their helpful and encouraging feedback.

Impressive amount of measures. Unfortunate that there were not more participants.

We agree that a higher number of participants would have enabled some great additional analyses, but would also like to emphasize that this study 1) has one of the largest sample sizes of breathwork studies to date (which to be fair have been chronically underpowered), and 2) has a sufficient sample size to confidently establish all the main effects presented here (e.g. changes in CO_2 levels and subjective experience, as well as their mutual relation).

Please **add effect sizes**, ideally Cohen's d, even when effects are not significant. The effect size can still be informative.
We thank the reviewer for pointing this out and have added Cohen's D values throughout the text, as well as in Supplementary Table S5.

I wish that the two conditions **could have been the same length,** but maybe that was impossible because there are clearly set session lengths for these two types of breathing exercises.

We agree with the reviewer that conducting both breathwork interventions over the same duration would have removed possible sources of variance. On the other hand, conducting both breathwork styles in the way they are most commonly practiced also gives our measurements an ecological validity that we see as crucial. Finally, the fact that both breathwork interventions have such similar short- and long-term effects despite the noticeable difference in duration also opens up some fascinating and practically relevant new insights: It suggests that longer sessions do not equate to deeper experiences of more intense long-term changes. In our view, this is a fascinating piece of information, because it not only narrows the field of potential underlying mechanisms, but also has direct implications for practical recommendations, in the sense that longer sessions do not seem to be needed in order for breathwork to be effective. We have now added this interpretation of our data in Lines 934-941 of the Discussion, subsection *Comparison between breathwork styles*.

It would have been better if there had been another score for when participants could not respond instead

of giving them the highest score in the altered states of consciousness scale. I think actively saying you are a 5 on that scale and not responding to the question are not necessarily the same thing. I do not see this as a fatal flaw because the authors also have the physiological measures.

We thank the reviewer for pointing this out, and have added this point to the discussion as a recommendation for future studies.

References

Francis, Gregory, and Evelina Thunell. 2021. 'Reversing Bonferroni'. *Psychonomic Bulletin & Review* 28 (3): 788–94. https://doi.org/10.3758/s13423-020-01855-z.

Ludbrook, John. 1991. 'On Making Multiple Comparisons in Clinical and Experimental Pharmacology and Physiology'. *Clinical and Experimental Pharmacology and Physiology* 18 (6): 379–92. https://doi.org/10.1111/j.1440-1681.1991.tb01468.x.

Mathur, Maya, and Tyler J. VanderWeele. 2018. 'New Metrics for Multiple Testing with Correlated Outcomes'. OSF. https://doi.org/10.31219/osf.io/k9g3b.

Midway, Stephen, Matthew Robertson, Shane Flinn, and Michael Kaller. 2020. 'Comparing Multiple Comparisons: Practical Guidance for Choosing the Best Multiple Comparisons Test'. *PeerJ* 8 (December):e10387. https://doi.org/10.7717/peerj.10387.

Narum, Shawn R. 2006. 'Beyond Bonferroni: Less Conservative Analyses for Conservation Genetics'. *Conservation Genetics* 7 (5): 783–87. https://doi.org/10.1007/s10592-005-9056-y.
